# Sn and Ge Complexes with Redox-Active Ligands as Efficient Interfacial Membrane-like Buffer Layers for p-i-n Perovskite Solar Cells

**DOI:** 10.3390/membranes13040439

**Published:** 2023-04-17

**Authors:** Azat F. Akbulatov, Anna Y. Akyeva, Pavel G. Shangin, Nikita A. Emelianov, Irina V. Krylova, Mariya O. Markova, Liliya D. Labutskaya, Alexander V. Mumyatov, Egor I. Tuzharov, Dmitry A. Bunin, Lyubov A. Frolova, Mikhail P. Egorov, Mikhail A. Syroeshkin, Pavel A. Troshin

**Affiliations:** 1 Federal Research Center of Problems of Chemical Physics and Medicinal Chemistry, Russian Academy of Sciences, Academician Semenov Ave. 1, Chernogolovka 142432, Russia; 2 N.D. Zelinsky Institute of Organic Chemistry, Russian Academy of Sciences, Moscow 119991, Russia; 3 Faculty of Technology of Inorganic Substances and High Temperature Materials, Dmitry Mendeleev University of Chemical Technology of Russia, Moscow 125047, Russia; 4 A.P. Nelyubin Institute of Pharmacy, Sechenov First Moscow State Medical University, Moscow 119991, Russia; 5 A.N. Frumkin Institute of Physical Chemistry and Electrochemistry, Russian Academy of Sciences, Moscow 119071, Russia

**Keywords:** tin, germanium, coordination compounds, cyclic voltammetry, UV–Vis spectroscopy, fluorescence spectroscopy, buffer layers, perovskite solar cells

## Abstract

Inverted perovskite solar cells with a p-i-n configuration have attracted considerable attention from the research community because of their simple design, insignificant hysteresis, improved operational stability, and low-temperature fabrication technology. However, this type of device is still lagging behind the classical n-i-p perovskite solar cells in terms of its power conversion efficiency. The performance of p-i-n perovskite solar cells can be increased using appropriate charge transport and buffer interlayers inserted between the main electron transport layer and top metal electrode. In this study, we addressed this challenge by designing a series of tin and germanium coordination complexes with redox-active ligands as promising interlayers for perovskite solar cells. The obtained compounds were characterized by X-ray single-crystal diffraction and/or NMR spectroscopy, and their optical and electrochemical properties were thoroughly studied. The efficiency of perovskite solar cells was improved from a reference value of 16.4% to 18.0–18.6%, using optimized interlayers of the tin complexes with salicylimine (1) or 2,3-dihydroxynaphthalene (2) ligands, and the germanium complex with the 2,3-dihydroxyphenazine ligand (4). The IR s-SNOM mapping revealed that the best-performing interlayers form uniform and pinhole-free coatings atop the PC_61_BM electron-transport layer, which improves the charge extraction to the top metal electrode. The obtained results feature the potential of using tin and germanium complexes as prospective materials for improving the performance of perovskite solar cells.

## 1. Introduction

Coordination compounds play an important role in biology and physiology and are commonly used in medicine and catalysis, in metallurgy for the extraction and processing of elements, and in analytical chemistry; they also are applied as materials for microelectronics and photovoltaics technologies [1,2,3,4,5]. The aforementioned applications mainly involve transition metal complexes, whereas the use of main-group-element coordination compounds are also becoming more widespread [6,7]. The main group elements usually do not have multiple stable oxidation states, unlike transition metals, but this issue could be mitigated by the use of redox-active ligands [8].

The Group 14 elements are of great importance in materials science, covering the historical gap from the first semiconductors to the currently emerging perovskite solar cells. In the series “silicon–germanium–tin–lead”, the germanium coordination compounds are more resistant to hydrolysis than the silicon derivatives [9]. In turn, the tin compounds are significantly less toxic compared to the lead-based analogs [10,11]. In that context, germanium compounds occupy a special position in coordination chemistry, since their toxicity is extremely low [12,13]. Thus, germanium- and tin-based materials can have an important competitive advantage over lead compounds and complexes of transition metals, which are generally toxic.

Tin coordination compounds with redox-active ligands are characterized by a relatively small HOMO-LUMO gap, which sometimes enables semiconductor properties, makes them fluorescent in the visible range, etc. [14]. They can be used as materials for optoelectronic devices [15], organic light-emitting diodes [16], and solar cells [17], as well as for fluorescence bioimaging [18,19]. Much less information can be found on the use of germanium coordination compounds in materials science. Still, it is already known that the use of redox-active ligands enables a significant decrease in the energy of LUMO levels of the compounds [20,21] and increase in their HOMO energy levels [22], thus delivering materials with a HOMO-LUMO gap of about 2 eV [22]. In addition, there was a report on the germanium derivative with phenanthrenediol and bipyridine that exhibited intense fluorescence in the visible range [23].

The practical application of Group 14 elements for energy harvesting is now greatly inspired by the emerging perovskite solar cells, which are based on complex lead and/or tin halides as absorber materials. Among different device architectures reported to date, p-i-n perovskite solar cells have many advantages, such as simple fabrication, high stability, and negligibly low hysteresis [24]. However, the interface between the top metal electrode (such as Ag and Al) and the electron transport layer (ETL, commonly represented by a fullerene derivative PC_61_BM) is not sufficiently stable, causing multiple aging effects, such as corrosion or delamination; the barrier formed between the ETL and top metal electrode leads to poor electron extraction [25]. Interface engineering can significantly enhance the performance and stability of p-i-n perovskite solar cells. It can be used to align material energy levels across the interface and to decrease interfacial losses caused by surface recombination [26]. Interface engineering can also improve the morphology of the electron transport layer, tailor the work function of contact layers, and enhance the long-term stability of the devices [25].

To date, many different materials have been investigated as interlayers in p-i-n perovskite solar cells, e.g., organic molecules [27], metal–organic frameworks [28], metal oxides [29], polymers [30], and carbon-based materials [31]. In particular, small organic molecules have attracted a particular interest due to their easy synthesis, purification, and tailorable and reproducible properties. Furthermore, organic interlayers can be deposited by solution-processing at low temperatures, which makes interface engineering a low-energy and inexpensive technology [32].

**Scheme 1:**
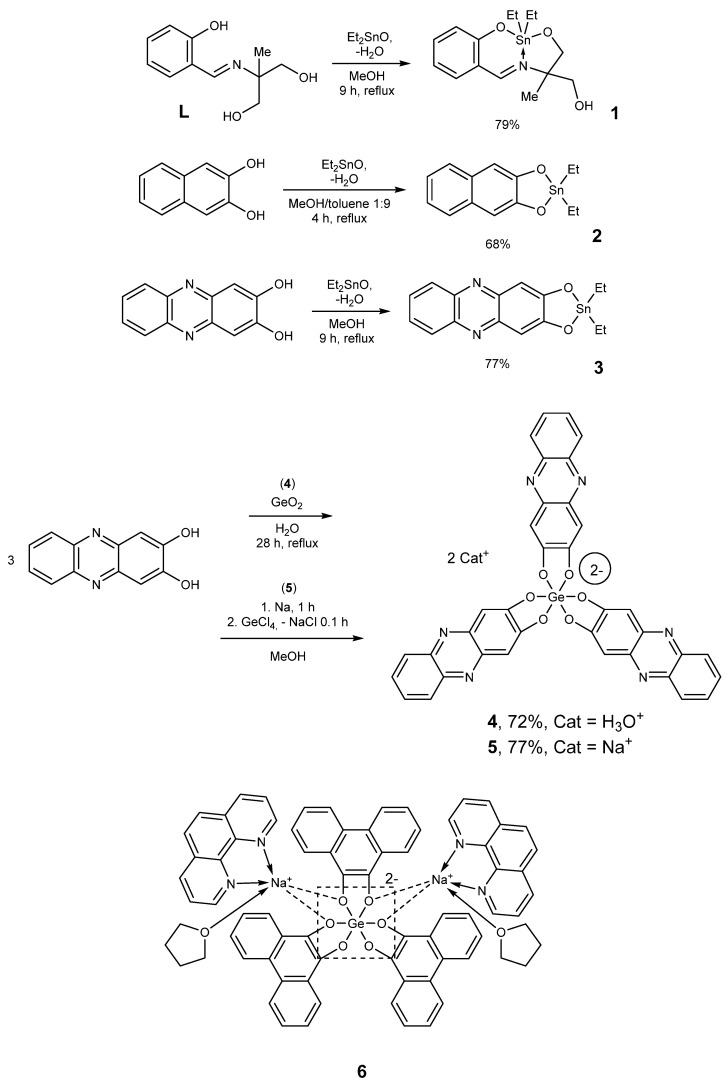
The synthesis of new tin and germanium coordination compounds **1**–**5** and also compound **6** previously reported by M. Nanjo et al. [23].

In this work, we present the synthesis of the tin and germanium coordination compounds **1**–**5** with the redox active dihydroxynaphthalene, dihydroxyphenazine, and Schiff base ligands (Scheme 1); investigation of their electrochemical behavior and optical properties; and evaluation of the designed materials buffer layers in inverted perovskite solar cells.

## 2. Materials and Methods

### 2.1. Materials

Diethyltin oxide Et_2_SnO was purchased from Gelest, germanium dioxide GeO_2_ was purchased from Germanium and Applications Ltd. (Novomoskovsk, Russia), and 2,3-dihydroxynaphtalene was from Acros Organics (Geel, Belgium). The methanol and toluene used in the synthesis were purified by standard methods [33]. For cyclic voltammetry, UV–Vis, and fluorescence experiments, dimethylformamide (Acros Organics, Geel, Belgium) with an initial water content of <100 ppm was stored in a glove box over dried 4 Å molecular sieves. Tetrabutylammonium tetrafluoroborate (Aldrich, Buchs, Switzerland) was dried under oil-pump vacuum at 80 °C for 4 h. After this, the water content in pure DMF and 0.1 M Bu_4_NBF_4_/DMF did not exceed 20 ppm, as determined by Karl Fischer titration.

### 2.2. Synthesis of Compounds ***1***–***6***

*(2,2-Diethyl-5-methyl-4,5-dihydrobenzo[h][1,3,6,2]dioxazastannonin-5-yl)methanol (compound **1**).* 2-((2-Hydroxybenzylidene)amino)-2-methylpropane-1,3-diol (synthesized according to [34]) (0.125 g, 0.597 mmol) was dissolved in 10 mL of methanol upon heating for 1 h, then Et_2_SnO (0.1144 g, 0.59 mmol) was added, and the reaction mixture was heated at reflux for 9 h. Methanol was distilled off and replaced with toluene. After cooling, the precipitate was filtered off and dried in a vacuum of a water jet pump. The yield of the product was 79% (0.181 g, 0.47 mmol).

^1^H NMR (300 MHz, DMSO-d_6_, δ, ppm): 8.35 (s, 1H, *J*_Sn-H_ = 45.58 Hz, CH=N), 7.40–7.36 (m, 1H, arom. CH), 7.31–7.25 (m, 1H, arom. CH), 6.63–6.58 (m, 2H, arom. CH), 5.15 (m, 1H, OH), 3.97–3.78 (m, 1H, CH_2_), 3.47–3.42 (m, 3H, CH_2_ and H of CH_2_), 1.39–0.99 (m, 13H, CH_3_ and two Et groups). ^13^C NMR (75 MHz, DMSO-d_6_, δ, ppm): 171.45 (CH=N), 169.38 (C), 136.01 (arom. CH), 135.63 (arom. CH), 121.41 (arom. CH), 117.56 (C), 115.05 (arom. CH), 69.56 (CH_2_), 64.56 (CH_2_), 19.11 (CH_3_), 13.30 (CH_2_ of Et), 12.20 (CH_3_ of Et), 9.84 and 9.78 (CH_3_ and CH_2_ of Et). HRMS for positive ions: found 386.0777 for [M + H]^+^, calculated 386.0775 (Supplementary Figures S3–S5).

*2,2-Diethylnaphtho[2,3-d][1,3,2]dioxastannole (compound **2***). Et_2_SnO (0.128 g, 0.66 mmol) was added to a mixture of 2,3-dihydroxynaphtalene (0.105 g, 0.66 mmol) in 2 mL of methanol and 18 mL of toluene. The reaction mixture was heated at reflux for 4 h and filtered. The precipitate was dried in vacuum. The yield of the product was 68% (0.15 g, 0.45 mmol).

^1^H NMR (300 MHz, DMSO-d_6_, δ, ppm): 7.38–7.33 (m, 2H, arom. CH), 6.99–6.94 (m, 2H, arom. CH), 6.77 (s, 2H, arom. CH), 1.35–1.27 (m, 4H, CH_2_), 1.17–1.12 (m, 6H, CH_3_); ^13^C NMR (75 MHz, DMSO-d_6_, δ, ppm): 155.97 (C-O), 128.60 (C), 124.52, 120.67, 106.18, 18.40 (CH_2_), 9.25 (CH_3_). HRMS for positive ions: found 337.0244 for [M + H]^+^, calculated 337.0247; found 359.0062 for [M+Na]^+^, calculated 359.0067 (Supplementary Figures S6–S9).

*2,2-Diethyl-[1,3,2]dioxastannolo[4,5-b]phenazine (compound **3**)* was prepared from 2,3-dihydroxyphenazine (synthesized according to [35]) and Et_2_SnO in a yield of 77% in a similar manner as that used for compound **1**.

^1^H NMR (300 MHz, DMSO-d_6_, δ, ppm): 7.93–7.89 (m, 2H, arom. CH), 7.56–7.53 (m, 2H. arom. CH), 6.85 (s, 2H, arom. CH), 1.38 (m, 4H, CH_2_), 1.15 (m, 6H, CH_3_). ^13^C NMR (75 MHz, DMSO-d_6_, δ, ppm): 162.83 (C), 143.35 (C), 139.88 (C), 127.78 (arom. CH), 126.22 (arom. CH), 103.08 (arom. CH), 20.41 (CH_2_), 9.37 (CH_3_). HRMS for positive ions: found 389.0303 for [M+H]^+^, calculated 389.0309 (Supplementary Figure S10–S13).

*Complex **4**.* 2,3-Dihydroxyphenazine (0.636 g, 3 mmol) and GeO_2_ (0.104 g, 1 mmol) were added to 20 mL of water. The reaction mixture was heated at reflux for 28 h and filtered. The precipitate was dried in vacuum and then extracted with chloroform. The yield of the product was 72% (0.505 g, 0.72 mmol).

^1^H NMR (300 MHz, DMSO-d_6_, δ, ppm): 8.2 (dd, 6H, J_1_ = 6.5 Hz, J_2_ = 3.4 Hz), 7.94 (dd, 6H, J_1_ = 6.5 Hz, J_2_ = 3.4 Hz), 7.26 (s, 6H). HRMS for negative ions: found 705.0579 for [M + H]^−^, calculated 705.0592 (Supplementary Figures S14 and S15).

*Complex **5**.* 2,3-Dihydroxyphenazine (0.636 g, 3 mmol) was dissolved in 50 mL of methanol. Then metallic sodium (0.138 g, 6 mmol) was added to the reaction mixture. The mixture was stirred for 1 h, under argon, and then GeCl_4_ (0.214 g, 1 mmol) was injected (the mixture color changed from red to green within several seconds). The precipitate of NaCl was filtered off, and then the solvent was removed from the filtrate in vacuum. The yield of the product was 77% (0.575 g, 0.77 mmol).

^1^H NMR (300 MHz, DMSO-d_6_, δ, ppm): 8.17 (dd, 6H, J_1_ = 6.5 Hz, J_2_ = 3.4 Hz), 7.91 (dd, 6H, J_1_ = 6.5 Hz, J_2_ = 3.4 Hz), 7.21 (s, 6H). HRMS for negative ions: found 705.0597 for [M+H]^−^, calculated 705.0592 and 352.0260 for [M]^2−^, calculated 352.0259 (Supplementary Figures S16–S18).

*Complex **6*** was synthesized according to [23].

### 2.3. Instrumentation

^1^H NMR (300 MHz) and ^13^C NMR (75 MHz) spectra were recorded in DMSO-d_6_ on a Bruker AM300 instrument at ambient temperature. NMR spectra were assigned using residual DMSO-d_6_ (^1^H NMR *δ* = 2.5 ppm, ^13^C NMR *δ* = 39.5 ppm). High-resolution mass spectra (HRMS) were measured on a Bruker micrOTOF II instrument, using electrospray ionization (ESI). The measurements were performed in the positive or negative ion mode (interface capillary voltage 4.5 kV), with a mass range from *m*/*z* 50 to *m*/*z* 1600, and external or internal calibration was performed with ESI Tuning Mix, Agilent. Syringe injection was used for solutions in acetonitrile or methanol (flow rate 3 μL min^−1^). Nitrogen was applied as a drying gas (flow rate 4 L min^−1^); the interface temperature was set at 200 °C. A Mettler-Toledo Titrator C10SD instrument was used for the Karl Fischer titration.

The oxidation and reduction behavior of the starting ligands and complexes **1**–**5** was analyzed by cyclic voltammetry, using an IPC-Pro-MF digital potentiostat (Econix). Solutions of the compounds were prepared, and all measurements were performed in an argon-filled glove box with water and oxygen levels below 1 ppm. The studied compounds that were dissolved in the supporting electrolyte (0.1 M Bu_4_NBF_4_/DMF) with a concentration of 3 × 10^−3^ M were electrochemically tested in a standard three-electrode glass cell at a potential scan rate of 0.1 V s^−1^. The working electrode was a glassy carbon disc electrode with a diameter of 1.7 mm. Before using, the electrode was polished with sandpaper and then with the GOI paste until the surface attained a mirror shine. The counter electrode was a Pt wire pre-annealed in a gas-burner flame to remove oxides and other possible contaminations. The potentials of the studied processes were measured versus the Ag wire coated with AgCl (prepared by galvanostatic anodizing in a 5% HCl solution) separated from the bulk electrolyte solution by an electrolytic bridge filled with the supporting electrolyte. The reference electrode was calibrated with respect to the ferrocene/ferrocenium couple.

UV–Vis spectroscopy was performed using an Agilent 8453 instrument. The spectra were registered for 1 × 10^−4^ M solutions in MeCN in a 10 mm quartz cell with a PTFE stopper. The solutions of the compounds were prepared in the same glove box. Before taking the cell out of the box, the cell–stopper contact was sealed with Parafilm, and then the spectrum was recorded within several minutes.

Fluorescence spectra were recorded using a Jasco FP-8300 spectrofluorometer. The technique for preparing samples and solutions was similar to that used in recording UV–Vis spectra. The quantum yields were determined using rhodamine B as a standard, whose quantum yield (0.40) in methanol is known [36].

Each CV curve and spectrum were reproduced at least three times.

### 2.4. X-ray Crystallographic Data and Refinement Details

X-ray diffraction data for **1** were collected at 100K on a Rigaku Synergy S diffractometer equipped with a HyPix6000HE area-detector (kappa geometry, shutterless ω-scan technique), using monochromatized Mo K_α_-radiation. The intensity data were integrated and semi-empirically corrected for absorption and decay by the CrysAlisPro program [37]. The structure was solved by direct methods, using SHELXT [38], and refined on *F*^2^, using SHELXL-2018 [39] in the OLEX2 program [40]. All non-hydrogen atoms were refined with individual anisotropic displacement parameters. The position of the hydroxy H3A atom was found from the electron density-difference map. All other hydrogen atoms were placed in ideal calculated positions (C-H distance = 0.950 Å for aromatic, 0.980 Å for methyl, and 0.990 Å for methylene hydrogen atoms) and refined as riding atoms with relative isotropic displacement parameters taken as *U*_iso_(H) = 1.5*U*_eq_(C) for methyl groups and *U*_iso_(H) = 1.2*U*_eq_(C) otherwise. A rotating group model was applied for methyl groups. Disorders were refined in a regular manner by applying similarity constraints on anisotropic displacement parameters on similar atoms and by constraining similar distances. The *SHELXTL* program suite [41] and the *Mercury* program [42] were used for molecular graphics in the Supplementary Materials and in the manuscript, correspondingly. Crystal data, data collection, and structure refinement details are summarized in Supplementary Table S1. The structure was deposited at the Cambridge Crystallographic Data Center, with the reference CCDC number 2243131; it also contains the supplementary crystallographic data. These data can be obtained free of charge from the CCDC via https://www.ccdc.cam.ac.uk/structures/ (accessed on 10 March 2023).

### 2.5. Device Fabrication and Characterization

Glass/ITO substrates (15 Ohm/sq., Kintec, Shenzhen, China) were sequentially cleaned with toluene and acetone and then sonicated in deionized water, acetone, and isopropanol. A solution of 1.5 mg/mL of poly[bis(4-phenyl)(4-methylphenyl)amine] (PTA) was spin-coated onto ITO at 4000 rpm for 20 s. The resulting PTA films were then dried at 100 °C for 10 min. The MAPbI_3_ precursor solutions (DMF:NMP = 4:1) were spin-coated at 4000 rpm, and toluene (150 mL) was dropped on the film 11 s after the initiation of spin-coating to induce the film crystallization. The spinning was continued for 60 s, and then the deposited films were annealed on a hotplate at 100 °C for 5 min. A 30 mg/mL solution of PC_61_BM in chlorobenzene was spin-coated at 1500 rpm on the top of the MAPbI_3_ films. The interfacial buffer layers of compounds **1**–**6** were deposited by spin-coating their isopropanol solutions (optimal concentrations are given below) at 4000 rpm in air. Aluminum top electrodes (100 nm) were deposited by thermal evaporation in high vacuum (10^−6^ mbar). The device active area was 0.08 cm^2^, as determined by a shadow mask. All steps of the device fabrication, except substrate cleaning and coating of compounds **1**–**6**, were carried out under an inert atmosphere inside a nitrogen glove box.

The current–voltage (*J*–V) characteristics of the devices were obtained under an inert atmosphere, using the simulated 100 mW/cm^2^ AM1.5 solar irradiation provided by a KHS Steuernagel solar simulator integrated with an MBraun glove box. The intensity of illumination was checked every time before each measurement, using a calibrated silicon diode with the known spectral response. The *J*–V curves were recorded using Advantest 6240A source-measurement units. The obtained *J_SC_* values were reconfirmed by integrating the EQE spectra against the standard AM1.5G spectrum. The EQE spectra were recorded under an inert atmosphere inside a nitrogen glove box, using a specially designed setup (LOMO instruments (Russia) and electric components from Stanford Research Instruments (USA)).

## 3. Results and Discussion

### 3.1. Synthesis of Compounds ***1***–***6***

**Figure 1:**
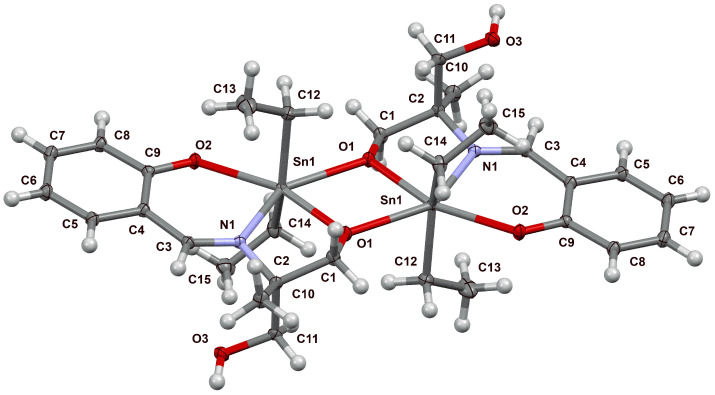
Molecular structure of **1** (*p* = 50%). Selected bond lengths (Å) and bond angles (°): Sn(1)-O(2) 2.2657(10), Sn(1)-O’(1) 2.4090(10), Sn(1)-O(1) 2.0992(10), Sn(1)-N(1) 2.2369(11), O(2)-Sn(1)-O’(1) 133.29(3), O(1)-Sn(1)-O(2) 157.64(4), O(1)-Sn(1)-O’(1) 69.07(4), Sn(1)-O(1)-Sn’(1) 110.93(4), O(1)-Sn(1)-N(1) 76.33(4), N(1)-Sn(1)-O(2) 81.34(4), and N(1)-Sn(1)-O’(1) 145.23(4).

One of the most common types of complexones used in the synthesis of coordination compounds is Schiff bases, among which 2-((2-hydroxybenzylidene)amino)-2-methylpropane-1,3-diol is often used (Scheme 1, **L**). At present, the complexation of **L** with iron [43], cobalt [43,44,45], nickel [43], copper [43], manganese [34,44], molybdenum [46,47], zinc [48], vanadium [49,50], etc., has been described. To the best of our knowledge, only one derivative of **L** with the main group element, germanium, has been published [51]. In the present work, we synthesized the tin complex **1** by the treatment of **L** with Et_2_SnO in methanol (Scheme 1). As can be seen from the X-ray diffraction data (Figure 1; Supplementary Figures S1 and S2 and Tables S1–S4), the target compound **1** contains the donor–acceptor bond with the imine nitrogen, in addition to the four Sn-C and Sn-O covalent bonds. It is known [52] that this bond can be much shorter in the tin complexes with Schiff bases than the typical Sn ← N coordination bond (>2.37 Å [53]). In particular, its length is 2.146–2.233 Å for the derivatives studied in [52] and 2.136–2.157 Å in [18]. This value is slightly longer for **1** (2.2369 Å) but also follows this trend. In addition, the bond length is quite close to the structurally similar motif containing two methyl substituents on tin [54] (2.230 Å). In a single crystal, compound **1** is organized into characteristic [55,56] dimers formed by two donor–acceptor bonds between the tin atoms and alcoholic oxygen atoms of the neighboring molecule. The Sn(1)-O(1) and Sn(1)-O’(1) bond lengths are 2.0992 and 2.4090 Å, respectively, and the angles O(1)-Sn(1)-O’(1) and Sn(1)-O(1)-Sn’(1) are 69.07° and 110.93°, resulting in a flat parallelogram. The ^1^H NMR (nuclear magnetic resonance), ^13^C NMR, and HRMS (high-resolution mass spectrometry) spectra of compound **1** are presented in Supplementary Figures S3–S5.

One of the most common types of redox-active ligands is represented by the catechol derivatives [57]. Having one aromatic ring and two sufficient donor oxygen substituents, catechol can be oxidized relatively easy, but the reduction of such systems is difficult. To mitigate that issue, one could increase the size of the aromatic system. Naphthalene can be considered the simplest redox-active motif with accessible reduction and oxidation potentials [58], and it is also capable of fluorescence with a relatively high quantum yield [59]. Thus, we also synthesized a new tin complex **2** by refluxing Et_2_SnO with 2,3-dihydroxynaphthalene in a methanol/toluene mixture, as described in the experimental section. It is worth noting that the Sn(IV) derivatives with dihydroxynaphthalene are poorly described in the literature [60,61], and their properties are insufficiently studied. The ^1^H NMR, ^13^C NMR, HSQC (heteronuclear single quantum coherence) NMR, and HRMS spectra of compound **2** are presented in Supplementary Figures S6–S9.

Phenazine is one of the most abundant structural motifs, both in practice [62] and in wildlife [63], enabling fluorescence in the visible range. The catechol derivative, 2,3-dihydroxyphenazine [64], was first utilized herein to obtain the tin complex **3** (^1^H NMR, ^13^C NMR, HSQC NMR, and HRMS spectra are shown in Supplementary Figures S10–S13). At the same time, when attempting to obtain germanium bis(catecholate) with such a ligand by its interaction with GeO_2_, i.e., similarly to how such a derivative was previously obtained with 2,3-dihydroxynaphthalene [22], it was found that complex **4**, with three ligands, was formed (^1^H NMR and HRMS shown in Supplementary Figures S14 and S15). The central part of the complex is the germanium-centered dianion, whereas the counterions are apparently two hydronium cations in the case of the synthesis in water under neutral conditions. If the synthesis is carried out with 2,3-dihydroxyphenazine sodium salt, the product is the complex **5** with sodium cations as counterions. The presence of the germanium dianion in the structure is confirmed by the ESI (electrospray ionization) HRMS data (Supplementary Figure S16), revealing the polyisotopic molecular ion with *m*/*z* 352.0260 (*z* = −2) characteristic of germanium. The ^1^H NMR and HRMS of compound **5** are presented in Supplementary Figures S17 and S18.

### 3.2. Optoelectronic and Physicochemical Properties of ***1***–***6***

The electrochemical behavior of the new compounds **1**–**5** upon oxidation and reduction was studied, and their absorption and fluorescence spectra were recorded. The results are summarized in Table 1.

**Figure 2:**
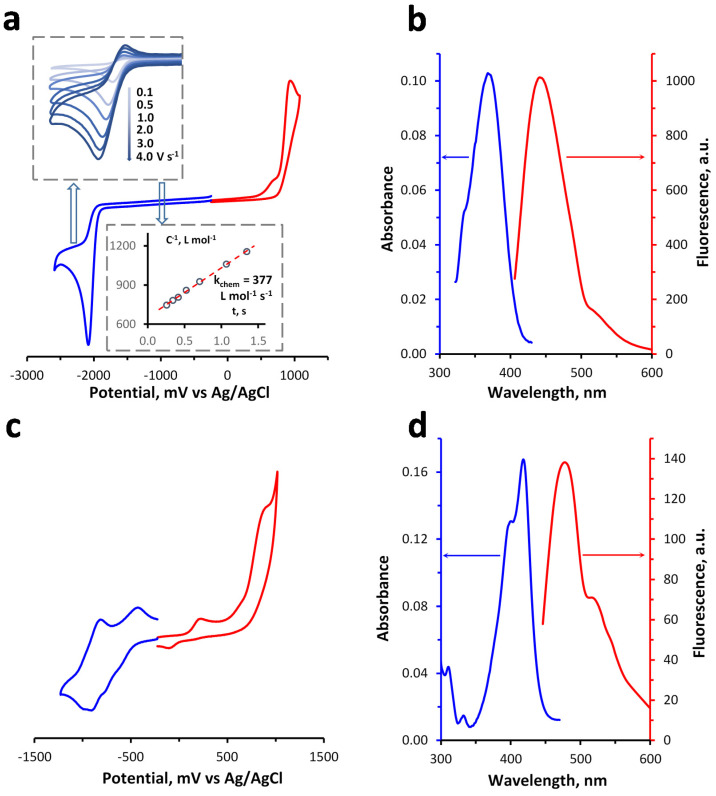
CV curves of the oxidation (red) and reduction (blue) of complexes **1** (**a**) and **4** (**c**) (C = 3 mM) in a 0.1 M Bu_4_NBF_4_/DMF supporting electrolyte on a glassy carbon disc electrode at a potential scan rate of 100 mV s^−1^. Insets show the reduction curves for **1** at various scan rates and second-order kinetic plot for the chemical reaction following the electrochemical reduction of **1**. Circles are experimental data points, whereas the dashed line is a linear fit. Absorption (blue) and fluorescence (red) spectra of **1** (**b**) and **4** (**d**) in DMF (C = 0.1 mM).

The cyclic voltammetry (CV) curves of **1** (Figure 2a) manifest irreversible reduction (–2.08 V) and oxidation (0.94 V) peaks. It should be noted that the reduction process becomes quasi-reversible with an increase in the potential scan rate. By analyzing [65] the CV curves recorded at the scan rates up to 4 V s^−1^, a second-order kinetic curve for the chemical reaction following the reduction was obtained. The reaction rate constant was determined as the slope of the kinetic curve: 377 L mol^−1^ s^−1^. The concentration of active radical anions was estimated as C_0_·I_p_^ox^/I_p_^red^ according to the Randles–Sevcik equation, assuming that the diffusion coefficients of **1** and radical anions were comparable (C_0_ is the concentration of **1** in the bulk solution; I_p_^red^ and I_p_^ox^ are the peak currents for the forward reduction and reverse oxidation peaks, respectively). According to the UV and fluorescence spectroscopy data, compound **1** shows its absorption maximum at 368 nm (Table 1) and fluorescence maximum (blue-violet light) at 443 nm (Figure 2b). This corresponds to the transition with an energy of 2.80 eV, which generally correlates with a potential difference of 3.02 V. The quantum yield of **1** is quite high and approaches 0.45.

Compound **2** is reduced at a very negative peak potential of –2.67 V close to the edge of the supporting electrolyte stability window. On the contrary, the oxidation of **2** proceeds quite easily, with a peak potential of 0.49 V. Both processes are chemically irreversible, which means that the radical cation and the radical anion of **2** are unstable. Using the oxidation and reduction peak potentials, the value of the HOMO/LUMO gap of **2** can be estimated as somewhat higher than 3 eV. The UV spectrum of **2** in DMF (Supplementary Figure S19) exhibits a maximum at 340 nm in the UV range close to the visible range (Table 1), while the compound shows violet-light emission with a maximum of 386 nm. The absorption and emission properties correspond to a radiative transition energy of 3.21 eV, which is quite close to the oxidation/reduction peak potential difference (3.16 V). The quantum yield of **2** is quite high and approaches 0.34, which is a notably higher value than, for example, that of naphthalene (0.23 [59]).

Compound **3** is a structural analogue of **2** with an enlarged aromatic system containing three conjugated rings and two heterocyclic nitrogen atoms, which enhance the acceptor properties of the system and modulate its chromophore behavior. Based on this, we expected compound **3** to be easier to reduce and harder to oxidize as compared to **2**. Indeed, as can be seen from the CV curves (Supplementary Figure S20), the reduction of compound **3** starts at −1.52 V, while it is oxidized at 0.86 V; i.e., the potential difference is 2.38 V (Table 1). The UV spectrum of **3** (Figure S20) shows an absorption maximum in the violet range at 419 nm, while the fluorescence is green, with a maximum at 527 nm. This corresponds to a radiative transition energy of 2.35 eV, which is quite close to the electrochemical potential difference of 2.38 V. The fluorescence behavior of **3** is characterized by a very high quantum yield of 0.84.

Compared to compound **3**, compound **4** contains more acceptor phenazine fragments, thus allowing us to expect a lower reduction potential and a higher oxidation potential. Indeed, compound **4** is irreversibly reduced at rather low potentials with the peak of −0.91 V (Figure 2c and Table 1). The irreversible peak at 0.91 V refers to the oxidation of **4**. According to fluorescence spectroscopy data (Figure 2d), compound **4** emits blue light with a maximum at 478 nm, which corresponds to a transition of 2.60 eV and poorly correlates with the electrochemical potential difference (1.82 V). The quantum yield of **4**, like that of **3**, is quite high and reaches 0.60.

The electrochemical behavior of compound **5** is similar to that of compound **4** since they are isostructural and differ only by the counterions (Scheme 1). Compound **5** is reduced and oxidized irreversibly at peak potentials of –0.81 V and 0.96 V, respectively (Supplementary Figure S21). However, the replacement of the counterion leads to a noticeable blue shift of the fluorescence maximum to 357 nm (3.48 eV, Table 1) and a decrease in the quantum yield down to 0.19.

We also investigated the thermal stability of compounds **1**–**6** by using thermal gravimetry technique (Supplementary Figure S22). It was found that compounds **1**–**3** are quite thermally stable, and their decomposition temperatures (5% weight loss) correspond to 254, 332, and 206 °C, respectively. Compound **1** decomposes in a single stage, most likely producing SnO_2_ (39% of weight) and volatile organic species. On the contrary, compound **2** decomposes in several stages, and the first one corresponds to the elimination of diethyl ether moiety (22% weight loss), whereas the solid residue apparently represents SnO (40% of remaining weight). Compound 3 undergoes a similar decomposition pathway involving an initial elimination of Et_2_O (19% weight loss) and then the rest of the organic ligands, thus leaving SnO in the residue. The lower decomposition temperature of **3**, as compared to that of **2**, could be related to the participation of the phenazine nitrogen atoms in stabilization of coordinationally unsaturated Sn ions, which are formed after the elimination of Et_2_O. Compounds **4**–**6** appeared to be much less thermally stable and have decomposition temperatures of 95, 137, and 195 °C, respectively. The observed lower stability of germanium complexes as compared to the tin derivatives might be partially related to the presence of volatile molecules, i.e., H_2_O in the case of **4** and tetrahydrofuran in the case of **6**. Still, it seems that the thermal stability of complexes is mainly influenced by the core element, which is Sn for **1**–**3** and Ge for **4**–**6**. Compounds **4** and **5** showed plateau-less decomposition behavior, whereas the weight of the non-volatile residue suggests that it has a composition of LGeO in the case of **4** (40% of weight) and LGeO*Na_2_O (48% of weight) in the case of **5** (L—dihydroxyphenazine ligand). Compound **6** undergoes decomposition with the main weight loss at 200–500 °C with the formation of Na_2_O*GeL_2_ (L—dihydroxyphenanthrene), which corresponds to ~39% of the residual weight. Further heating up to 1000 °C results in purely inorganic residue of Na_2_GeO_3_ (residual weight ~14%). To summarize, all compounds **1**–**6** showed sufficiently high thermal stability and should not decompose under the solar cell operational conditions.

### 3.3. Evaluation of the Complexes ***1***–***6*** as Buffer Layer Materials in p-i-n Perovskite Solar Cells

**Figure 3:**
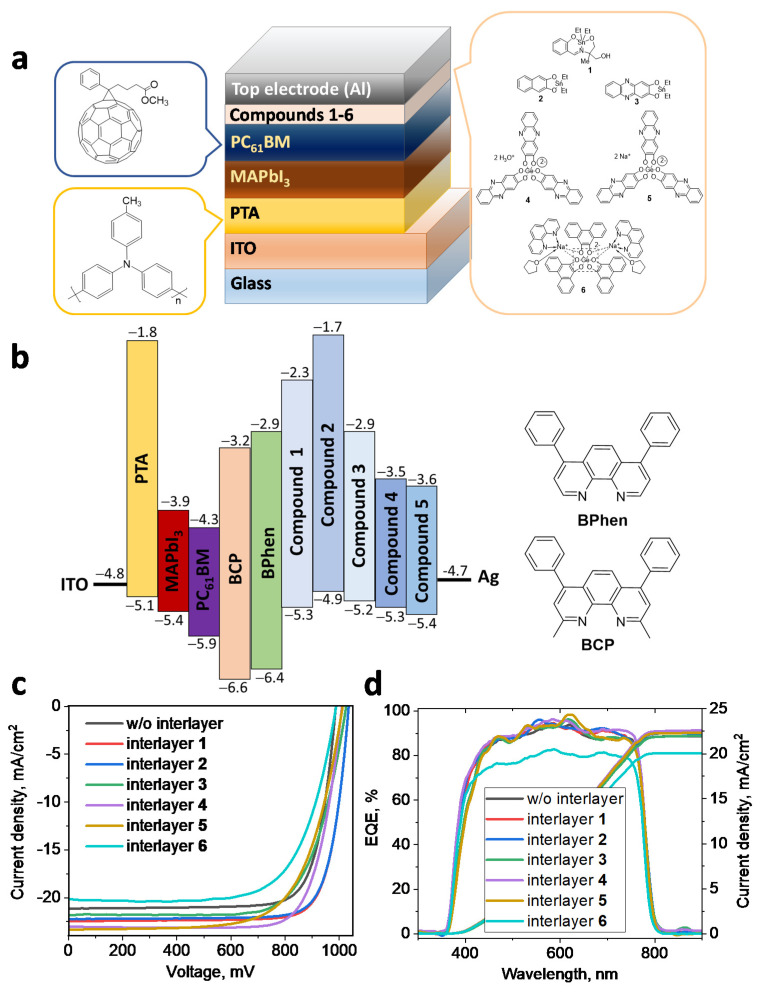
Schematic layout of the perovskite solar cell architecture, using complexes **1**–**6** as interlayer materials between PC_61_BM and Al (**a**); energy level diagram for used device components (**b**); *J*-V curves (**c**); and EQE spectra (**d**) of the best-performing solar cells, using **1**–**6**.

In order to evaluate the practical applicability of the synthesized materials, we investigated their performance as buffer layers at the ETL/top electrode interface in p-i-n perovskite solar cells. Figure 3a shows the schematic layout of the used device architecture with the glass/ITO/PTA/MAPbI_3_/PC_61_BM/Al configuration. Poly[bis(4-phenyl)(4-methylphenyl)amine] (PTA) and phenyl-C_61_-butyric acid methyl ester (PC_61_BM) were used as materials for hole- and electron-transport layers, respectively, whereas the MAPbI_3_ (where MA is methylammonium CH_3_NH_3_^+^) complex lead iodide with the perovskite lattice was used as an absorber material. The device structure was completed by the deposition of aluminum top electrodes, using the thermal evaporation of metal in high vacuum. All details of perovskite solar cell fabrication are given in the Materials and Methods.

Tin and germanium complexes **1**–**6** were used as interlayers between PC_61_BM and the Al top electrode. It should be noted that compounds **1**–**3** have considerably higher lowest unoccupied molecular orbital (LUMO) energies than PC_61_BM (Figure 3b). However, such cathode interlayer materials as bathophenanthroline (BPhen) and bathocuproine (BCP), which are commonly used in highly efficient PSCs, have similar energy levels. Thus, high LUMO energy levels do not prevent efficient electron extraction most probably due to the doping of these interlayers by metal deposited atop, which results in Fermi level pinning and forming an ohmic contact interphase between the ETL (PC_61_BM) and top metal electrode. We believe that compounds **1**–**6** could function in the same way as BCP and BPhen. Another important aspect is the ability of BCP and BPhen to chelate metal ions and prevent their diffusion deeper into the ETL structure. In that context, the complexes of tin and germanium studied in this work could also trap metal atoms/ions due to their interactions with redox-active ligands, also bearing chelating phenazine units.

The deposition of compounds **1**–**6** atop PC_61_BM films modified their surface properties: it increased their surface energy mainly due to the rise of its polar component (Table S5). The water contact angles decreased, particularly for PC_61_BM/**5** system (38.2°), in comparison with bare PC_61_BM films (91.1°). Thus, we could conclude that the deposition of compounds **1**–**6** makes PC_61_BM films more hydrophilic, which is favorable for the adhesion of top metal electrode layers forming stable interfaces with oxide/nitride passivation interlayers.

**Figure 4:**
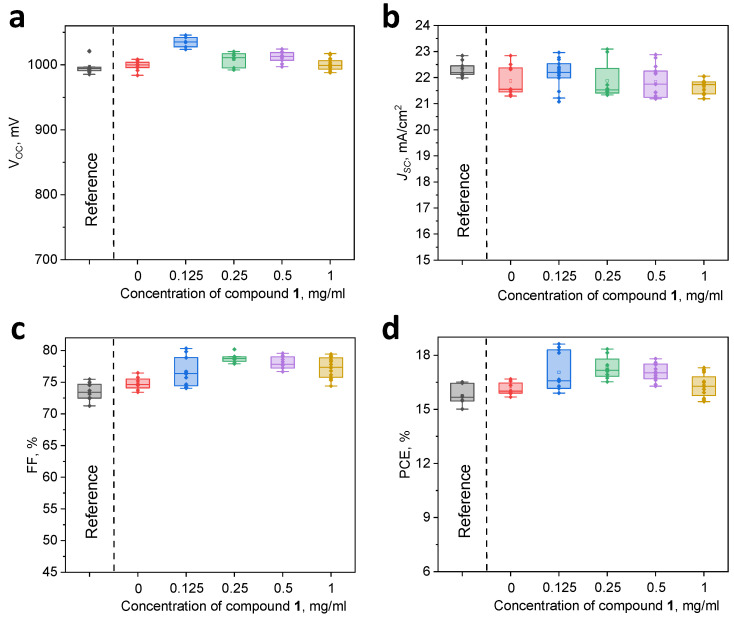
Open-circuit voltage, V_OC_ (**a**); short-circuit current density, *J*_SC_ (**b**); fill factor, FF (**c**); and power conversion efficiency, PCE (**d**), of perovskite solar cell as a function of the concentration of compound **1** in the solution used for films’ coating.

To investigate the behavior of compounds **1**–**6** as cathode interlayers, we deposited them on the top of the PC_61_BM layer by spin coating from isopropanol solutions at a constant spinning rate of 4000 rpm. In this experiment, we also assembled control samples of perovskite solar cells in which the PC_61_BM layer was exposed to pure isopropanol. To optimize the layer thickness of compounds **1**–**6**, we varied their concentrations in solution. The dependence of the solar cell parameters on material concentration is presented in Figure 4 for compound **1**: it is seen that the best device performance was achieved using 0.125 mg mL^−1^. Similar plots for other compounds are shown in Supplementary Figures S23–S28, whereas all numeric information is given in Supplementary Tables S6–S11. It should be noted that the maximal achievable concentration of compounds **1**–**6** was 1 mg/mL, so we could not go above it due to the solubility limit.

Figure 3c shows the current–voltage (*J*-V) characteristics of the best perovskite solar cells assembled using interlayers **1**–**6**, whereas their characteristics are summarized in Table 2. As one may see from these data, the use of compound **6** led to the significant deterioration of the photovoltaic parameters of the devices. At the same time, compounds **3** and **5** had almost no effect on the device performance and demonstrated power conversion efficiencies (PCE) close to those of the reference samples. Finally, complexes **1**, **2**, and **4** noticeably increased the solar cells efficiency from 16.4% to 18.6%, 18.5%, and 18.0%, respectively. This improvement is primarily associated with an increase in the fill factor, which may indicate a faster charge extraction and more efficient charge transport processes in the perovskite solar cells using these interlayers. To confirm the obtained short-circuit current density (*J*_SC_), we integrated the external quantum efficiency (EQE) spectra against the reference AM1.5G solar emission spectrum and obtained comparable current density values (Figure 3d).

It is notable that the lowest performance was observed for solar cells using compounds **5** (16.1%) and particularly **6** (13.9%) as interfacial buffer layer materials. The deterioration of the photovoltaic characteristics could be ascribed to the presence of mobile sodium ions in both **5** and **6**, which apparently lead to some undesired interfacial effects. Indeed, compound **4**, which differs from **5** just by the absence of sodium counter ions, enables a considerably better photovoltaic performance (18% for the best cells). It is highly possible that the deposition of aluminum atop **5** and **6** leads to some unfavorable interfacial chemical processes, e.g., top electrode corrosion mediated by sodium in 0 (metallic) or +1 oxidation states. The observed correlation suggests that sodium, and perhaps also other alkali metal ions, should be avoided in the composition of interfacial buffer-layer materials.

We also performed preliminary stability tests for the reference cells by using bare PC_61_BM as ETL and the devices assembled with PC_61_BM/compound **1** combination. Both types of devices expectedly showed almost identical degradation behavior (Supplementary Figure S29) since it is known that the operational stability of p-i-n perovskite solar cells with fullerene-based ETLs is limited by the absorber/ETL interface [66,67]. Thus, even though interlayers based on compounds **1**, **2**, and **4** can enhance the performance of p-i-n PSCs presumably due to the formation of optimal interface with the top metal contact, some additional absorber/ETL interface engineering is needed to achieve the desired device operational stability.

**Figure 5:**
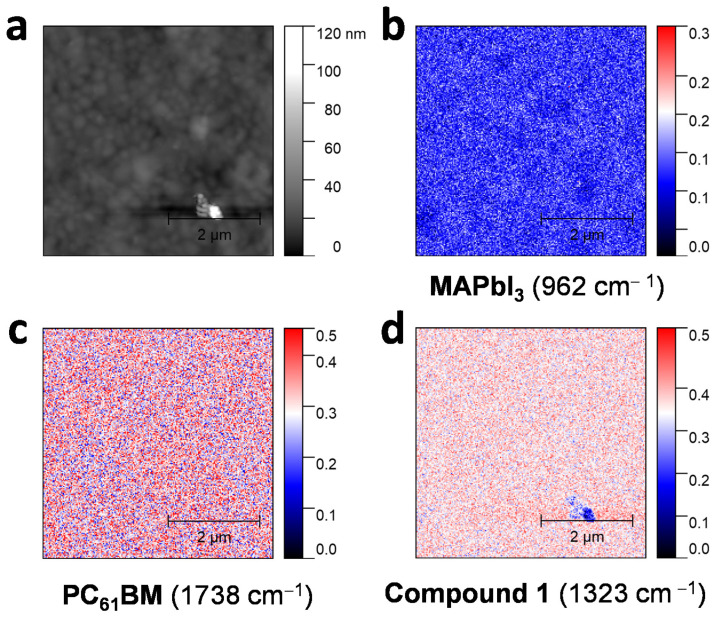
AFM topography of ITO/PTA/MAPbI_3_/PC_61_BM /**1** stack films (**a**) and the corresponding IR s-SNOM maps at the frequencies of 962 cm^−1^ (MAPbI_3_, (**b**)), 1738 cm^−1^ (PC_61_BM, (**c**)), and 1323 cm^−1^ (compound **1**, (**d**)).

To verify the formation of uniform buffer coatings atop PC_61_BM, we investigated the surface composition and morphology of the ITO/PTA/MAPbI_3_/PC_61_BM/(compound **1**–**6**) stacks by using the infrared scattering scanning near-field microscopy (IR s-SNOM) technique. The assembled perovskite solar cells were scanned in the areas between the top electrodes, thus enabling a direct comparison of the film morphology and photovoltaic performance characteristics. Basically, IR s-SNOM is used for mapping the distribution of a material at some characteristics IR absorption frequencies. Thus, considering the Fourier-transform infrared (FTIR) spectra of MAPbI_3_, PC_61_BM, and compound **1** (Supplementary Figure S30), non-overlapping peaks in vibrational spectra at 962 cm^−1^ (CH_3_NH_3_ group in MAPbI_3_), 1738 cm^−1^ (carbonyl group of PC_61_BM), and 1323 cm^−1^ (characteristic of **1**) were chosen for IR s-SNOM mapping. Figure 5 shows the exemplary set of AFM topography and IR s-SNOM images for the samples using compound **1** as interfacial coating, whereas similar graphs for other systems are presented in Supplementary Figures S31–S40.

It should be noted that the IR s-SNOM technique collects the information from the upper 10–15 nm of the films. Since the thickness of the interfacial buffer coatings of **1**–**6** was less than 15 nm, it is not surprising that the PC_61_BM underlayer was clearly visible on all the images. On the contrary, none of the samples showed the presence of pinholes or other types of defects, where the MAPbI_3_ perovskite phase is exposed on the surface. Thus, we could conclude that the electron transport layer of PC_61_BM in combination with the deposited above buffer layers of compounds **1**–**6** provide very uniform and defect-free coatings atop the perovskite absorber films.

Compounds **1**–**6** also showed a very homogeneous distribution over the PC_61_BM films’ surface, as revealed by IR s-SNOM microscopy (Figure 5 and Supplementary Figures S29–S38). Basically, there were no noticeable differences in the film morphology and the uniformity of distribution of compounds **1**–**6** in the studied samples, which could account for significant variations in the photovoltaic performance of these systems. Therefore, we could conclude that the performance of perovskite solar cells is affected mostly by the chemical nature of the buffer layers rather than the morphology of their films. Thus, this feature has to be addressed in further rational designs of interfacial coatings for p-i-n perovskite solar cells.

## 4. Conclusions

We designed and thoroughly characterized three new tin and two novel germanium complexes with redox-active ligands, which demonstrated interesting optical and electrochemical characteristics, in particular, high fluorescence quantum yields (up to 0.84) for green (527 nm for **3**) and even deep blue (386 nm for **2** and 357 nm for **5**) emission; this points toward potential applications in nanophotonics (LEDs and lasers). Herein, we systematically explored the synthesized coordination compounds as interfacial buffer layer materials for inverted p-i-n perovskite solar cells. All of these compounds formed uniform coatings above the PC_61_BM electron transport layer and underneath top metal electrode (Al) as revealed by IR s-SNOM mapping. It has been shown that introducing buffer layers of compounds **1**, **2**, and **4** at the PC_61_BM/Al interface results in a substantial increase in the device power conversion efficiency from 16.0% to 18.0–18.6%. Some correlations were revealed between the molecular structure of the compounds and their effect on the perovskite solar cell performance. In particular, the presence of Na^+^ counterions in complexes **5** and **6** resulted in deterioration of all device characteristics presumably due to unfavorable interfacial reactions (e.g., top electrode corrosion) promoted by alkali metal. We believe that this study provides useful design guidelines for developing efficient interfacial materials for high-performance perovskite solar cells.

## Figures and Tables

**Table 1:** Peak potentials for the oxidation and reduction of **1**–**5**, absorption and fluorescence maxima in DMF, Stokes shifts, and quantum yields.

Compound	E_p_^ox^, V *	ΔE, V *	λ_abs_, nm	λ_fl_, nm	Stokes Shift, cm^−1^	Φ_fl_
	E_p_^red^, V *		W_abs_, eV	W_fl_, eV		
**1**	0.936	3.019	368	443	4601	0.45
	−2.083		3.37	2.80		
**2**	0.490	3.155	340	386	3505	0.34
	−2.665		3.65	3.21		
**3**	0.858	2.379	419	527	4891	0.84
	−1.521		2.96	2.35		
**4**	≈0.908	≈1.817	418	478	3003	0.60
	≈−0.909		2.97	2.60		
**5**	≈0.956	≈1.762	342	357	1229	0.19
	≈−0.806		3.63	3.48		

* 0.1 M Bu_4_NBF_4_/DMF as a supporting electrolyte; glassy carbon electrode; scan rate, 100 mV s^−1^; potentials vs. Ag/AgCl.

**Table 2:** Characteristics of the solar cells, using derivatives **1**–**6** as interlayers (champion device characteristics are given in brackets).

Interlayer	Optimal Material Concentration, mg/mL	V_OC_, mV	J_SC_, mA/cm^2^	FF, %	PCE, %
none	-	994 ± 26 (1020)	22.2 ± 0.9 (23.1)	69 ± 3 (70)	15.8 ± 0.6 (16.4)
**1**	0.125	1000 ± 35 (1035)	22.0 ± 1.0 (22.5)	78 ± 2 (80)	17.6 ± 1.0 (18.6)
**2**	1	1035 ± 11 (1035)	22.2 ± 0.7 (22.2)	79 ± 1 (80)	18.0 ± 0.5 (18.5)
**3**	1	1012 ± 10(1022)	21.5 ± 0.5 (21.6)	71 ± 3 (74)	15.8 ± 0.5 (16.3)
**4**	1	1013 ± 11 (1010)	22.3 ± 0.7 (23.0)	75 ± 3 (77)	17.2 ± 0.8 (18.0)
**5**	0.25	998 ± 14 (1012)	22.9 ± 0.3 (23.2)	67 ± 2 (69)	15.6 ± 0.5 (16.1)
**6**	0.25	999 ± 19 (988)	20.3 ± 0.9 (20.1)	67 ± 4 (70)	13.2 ± 0.7 (13.9)

## Data Availability

The data presented in this study are available in the article and Supplementary Materials.

## Supplementary Materials

The following supporting information can be downloaded at https://www.mdpi.com/article/10.3390/membranes13040439/s1, Table S1: Crystal data, data collection and structure refinement details refinement for **1**; Figure S1: The molecular structure of **1** (*p* = 50%); Table S2: Selected bond lengths (Å) for **1**; Table S3: Selected bond angles (°) for **1**; Table S4: Hydrogen bonds for **1** (Å and °); Figure S2: Hydrogen bonding in **1**; Figure S3: ^1^H spectrum of compound **1**; Figure S4: ^13^C NMR spectrum of compound **1**; Figure S5: HRMS spectra of compound **1**; Figure S6: ^1^H NMR spectrum of compound **2**; Figure S7: ^13^C NMR spectrum of compound **2**; Figure S8: HSQC NMR spectrum of compound **2**; Figure S9: HRMS spectra of **2**; Figure S10: ^1^H NMR spectrum of compound **3**; Figure S11: ^13^C NMR spectrum of compound **3**; Figure S12: HSQC NMR spectrum of compound **3**; Figure S13: HRMS spectra of **3**; Figure S14: ^1^H NMR spectrum of compound **4**; Figure S15: HRMS spectrum of **4**; Figure S16: ESI-HRMS spectra (negative ion mode, MeOH) of the germanium dianion (*z* = 2) of **5**; Figure S17: ^1^H NMR spectrum of compound **5**; Figure S18: HRMS spectrum of **5**; Figure S19: CV curves of oxidation and reduction of **2** in a 0.1 M Bu_4_NBF_4_/DMF supporting electrolyte on a glassy carbon disc electrode at a potential scan rate of 100 mV s^−1^. Absorbance and fluorescence spectra of **2** in DMF; Figure S20: CV curves of oxidation and reduction of **3** in a 0.1 M Bu_4_NBF_4_/DMF supporting electrolyte on a glassy carbon disc electrode at a potential scan rate of 100 mV s^−1^. Absorbance and fluorescence spectra of **3** in DMF; Figure S21: CV curves of oxidation and reduction of **5** in a 0.1 M Bu_4_NBF_4_/DMF supporting electrolyte on a glassy carbon disc electrode at a potential scan rate of 100 mV s^−1^. Absorbance and fluorescence spectra of **5** in DMF; Figure S22: *J-V* curves and EQE spectra of perovskite solar cells with different concentrations of compound **1**; Table S5: Photovoltaic parameters of perovskite solar cells, using compound **1** as interlayer; Figure S23: V_OC_, J_SC_, FF, and PCE of PSCs as a function of concentration of **2**. *J*-V curves and EQE of the best devices; Table S6: Photovoltaic parameters of best solar cells, using of **2** as interlayer; Figure S24: V_OC_, J_SC_, FF, and PCE of PSCs as a function of concentration of **3**. I-V curves and EQE of the best devices; Table S7: Photovoltaic parameters of best solar cells, using of **3** as interlayer; Figure S25: V_OC_, J_SC_, FF, and PCE of PSCs as a function of concentration of **4**. I-V curves and EQE of the best devices; Table S8: Photovoltaic parameters of best solar cells, using of **4** as interlayer; Figure S26: V_OC_, J_SC_, FF, and PCE of PSCs as a function of concentration of **5**. I-V curves and EQE of the best devices; Table S9: Photovoltaic parameters of best solar cells, using of **5** as interlayer; Figure S27: V_OC_, J_SC_, FF, and PCE of PSCs as a function of concentration of **6**. I-V curves and EQE of the best devices; Table S10: Photovoltaic parameters of best solar cells, using of **6** as interlayer; Figure S28: ATR FTIR spectra of MAPbI_3_, PC_61_BM, and **1**; Figure S29: ATR FTIR spectra of MAPbI_3_, PC_61_BM, and **2**; Figure S30: AFM topography of ITO/PTA/MAPbI_3_/PC_61_BM/**2** film; mappings of ITO/PTA/MAPbI_3_/ PC_61_BM/**2** topography at frequencies of 962 cm^−1^, 1738 cm^−1^, and 1002 cm^−1^, which are characteristic for MAPbI_3_, PC_61_BM, and **2**, respectively; Figure S31: ATR FTIR spectra of MAPbI_3_, PC_61_BM and **3**; Figure S32: AFM topography of ITO/PTA/MAPbI_3_/PC_61_BM/**3** film; mappings of ITO/PTA/MAPbI_3_/PC_61_BM/**3** topography at frequencies of 1249 cm^−1^, 1738 cm^−1^, and 1519 cm^−1^, which are characteristic for MAPbI_3_, PC_61_BM, and **3**, respectively; Figure S33: ATR FTIR spectra of MAPbI_3_, PC_61_BM, and **4**; Figure S34: AFM topography of ITO/PTA/MAPbI_3_/ PC_61_BM/**4** film; mappings of ITO/PTA/MAPbI_3_/PC_61_BM/**4** topography at frequencies of 962 cm^−1^, 1738 cm^−1^, and 1146 cm^−1^, which are characteristic for **4**, PC_61_BM, and MAPbI_3_, respectively; Figure S35: ATR FTIR spectra of MAPbI_3_, PC_61_BM, and **5**; Figure S36: AFM topography of ITO/PTA/MAPbI_3_/PC_61_BM/**5** film; mappings of ITO/PTA/MAPbI_3_/PC_61_BM/**5** topography at frequencies of 962 cm^−1^, 1738 cm^−1^, and 1199 cm^−1^, which are characteristic for **5**, PC_61_BM, and MAPbI_3_, respectively; Figure S37: ATR FTIR spectra of MAPbI_3_, PC_61_BM, and **6**; Figure S38: AFM topography of ITO/PTA/MAPbI_3_/PC_61_BM/**6** film; mappings of ITO/PTA/MAPbI_3_/PC_61_BM/**6** topography at frequencies of 962 cm^−1^, 1738 cm^−1^, and 1586 cm^−1^, which are characteristic for **6**, PC_61_BM, and MAPbI_3_, respectively; Figure S39: ATR FTIR spectra of MAPbI3, PC61BM and 6; Figure S40: AFM topography of ITO/PTA/MAPbI3/PC61BM/6 film; mappings of ITO/PTA/MAPbI3/PC61BM/6 topography at frequencies of 962 cm^−1^, 1738 cm^−1^, and 1586 cm^−1^, which are characteristic for 6, PC61BM, and MAPbI3, respectively.

## References

[1] Haas K.L., Franz K.J. (2009). Application of Metal Coordination Chemistry to Explore and Manipulate Cell Biology. Chem. Rev..

[2] Srivastva A.N. (2020). Stability and Applications of Coordination Compounds.

[3] Sharma J., Dogra P., Sharma N., Ajay (2019). Applications of coordination compounds having Schiff bases: A review. AIP Conf. Proceed..

[4] Malinowski J., Zych D., Jacewicz D., Gawdzik B., Drzeżdżon J. (2020). Application of Coordination Compounds with Transition Metal Ions in the Chemical Industry—A Review. Int. J. Mol. Sci..

[5] Mohammed H.S., Tripathi V.D. (2020). Medicinal Applications of Coordination Complexes. J. Phys. Conf. Ser..

[6] Lichtenberg C. (2020). Main-Group Metal Complexes in Selective Bond Formations Through Radical Pathways. Chem. Eur. J..

[7] Morsali A., Hashemi L. (2017). Main Group Metal Coordination Polymers: Structures and Nanostructures.

[8] Nikolaevskaya E.N., Druzhkov N.O., Syroeshkin M.A., Egorov M.P. (2020). Chemistry of diazadiene type ligands with extra coordination groups. Prospects of reactivity. Coord. Chem. Rev..

[9] Roth D., Wadepohl H., Greb L. (2020). Bis(perchlorocatecholato)germane: Hard and Soft Lewis Superacid with Unlimited Water Stability. Angew. Chem. Int. Ed..

[10] Wang M., Wang W., Ma B., Shen W., Liu L., Cao K., Chen S., Huang W. (2021). Lead-Free Perovskite Materials for Solar Cells. Nano-Micro Lett..

[11] Liu X., Wu T., Luo X., Wang H., Furue M., Bessho T., Zhang Y., Nakazaki J., Segawa H., Han L. (2022). Lead-Free Perovskite Solar Cells with Over 10% Efficiency and Size 1 cm^2^ Enabled by Solvent–Crystallization Regulation in a Two-Step Deposition Method. ACS Energy Lett..

[12] Kadomtseva A.V., Mochalov G.M., Kuzina O.V. (2021). Biologically Active Coordination Compounds of Germanium. Synthesis and Physicochemical Properties. Russ. J. Org. Chem..

[13] Saverina E.A., Sivasankaran V., Kapaev R.R., Galushko A.S., Ananikov V.P., Egorov M.P., Jouikov V.V., Troshin P.A., Syroeshkin M.A. (2020). An environment-friendly approach to produce nanostructured germanium anodes for lithium-ion batteries. Green Chem..

[14] Takano K., Takahashi M., Fukushima T., Takezaki M., Tominaga T., Akashi H., Takagi H., Shibahara T. (2012). Fluorescent Tin(IV) Complexes with Schiff Base Ligands: Synthesis, Structures, and Fluorescence Lifetime. Bull. Chem. Soc. Jpn..

[15] Sánchez-Vergara M.E., Hamui L., Gómez E., Chans G.M., Galván-Hidalgo J.M. (2021). Design of Promising Heptacoordinated Organotin (IV) Complexes-PEDOT: PSS-Based Composite for New-Generation Optoelectronic Devices Applications. Polymers.

[16] Endo A., Ogasawara M., Takahashi A., Yokoyama D., Kato Y., Adachi C. (2009). Thermally Activated Delayed Fluorescence from Sn^4+^-Porphyrin Complexes and Their Application to Organic Light-Emitting Diodes—A Novel Mechanism for Electroluminescence. Adv. Mater..

[17] Cantón-Díaz A.M., Muñoz-Flores B.M., Moggio I., Arias E., León A.D., García-López M.C., Santillán R., Ochoa M.E., Jiménez-Pérez V.M. (2018). One-pot microwave-assisted synthesis of organotin Schiff bases: An optical and electrochemical study towards their effects in organic solar cells. New J. Chem..

[18] Jiménez-Pérez V.M., García-López M.C., Muñoz-Flores B.M., Chan-Navarro R., Berrones-Reyes J.C., Dias H.V.R., Moggio I., Arias E., Serrano-Mireles J.A., Chavez-Reyes A. (2015). New application of fluorescent organotin compounds derived from Schiff bases: Synthesis, X-ray structures, photophysical properties, cytotoxicity and fluorescent bioimaging. J. Mater. Chem. B.

[19] Lopez-Espejel M., Gomez-Trevino A., Munoz-Flores B.M., Treto-Suarez M.A., Schott E., Paez-Hernandez D., Zarate X., Jimenez-Perez V.M. (2021). Organotin Schiff bases as halofluorochromic dyes: Green synthesis, chemio-photophysical characterization, DFT, and their fluorescent bioimaging in vitro. J. Mater. Chem. B.

[20] Nikolaevskaya E.N., Saverina E.A., Starikova A.A., Farhati A., Kiskin M.A., Syroeshkin M.A., Egorov M.P., Jouikov V.V. (2018). Halogen-free GeO_2_ conversion: Electrochemical reduction vs. complexation in (DTBC)_2_Ge[Py(CN)_n_] (n = 0..2) complexes. Dalton Trans..

[21] Nikolaevskaya E.N., Shangin P.G., Starikova A.A., Jouikov V.V., Egorov M.P., Syroeshkin M.A. (2019). Easily electroreducible halogen-free germanium complexes with biologically active pyridines. Inorg. Chim. Acta.

[22] Shangin P.G., Krylova I.V., Lalov A.V., Kozmenkova A.Y., Saverina E.A., Buikin P.A., Korlyukov A.A., Starikova A.A., Nikolaevskaya E.N., Egorov M.P. (2021). Supramolecular D⋯A-layered structures based on germanium complexes with 2,3-dihydroxynaphthalene and N,N′-bidentate ligands. RSC Adv..

[23] Nanjo M., Yoneda T., Iwamatsu K. (2022). Hypercoordinate germanium complexes with phenanthrene-9,10-diolate ligands: Synthesis, structure, and electronic properties. Mendeleev Commun..

[24] Meng L., You J.B., Guo T.F., Yang Y. (2016). Recent Advances in the Inverted Planar Structure of Perovskite Solar Cells. Acc. Chem. Res..

[25] Yang J., Luo X., Zhou Y., Li Y., Qiu Q., Xie T. (2022). Recent Advances in Inverted Perovskite Solar Cells: Designing and Fabrication. Int. J. Mol. Sci..

[26] Bao X., Wang J., Li Y., Zhu D., Wu Y., Guo P., Wang X., Zhang Y., Wang J., Yip H.-L. (2017). Interface Engineering of a Compatible PEDOT Derivative Bilayer for High-Performance Inverted Perovskite Solar Cells. Adv. Mater. Interfaces.

[27] Liu L., Mei A., Liu T., Jiang P., Sheng Y., Zhang L., Han H. (2015). Well-Defined Thiolated Nanographene as Hole-Transporting Material for Efficient and Stable Perovskite Solar Cells. J. Am. Chem. Soc..

[28] Wu S., Li Z., Li M.-Q., Diao Y., Lin F., Liu T., Zhang J., Tieu P., Gao W., Qi F. (2020). 2D metal-organic framework for stable perovskite solar cells with minimized lead leakage. Nat. Nanotechnol..

[29] Jeng J.-Y., Chen K.-C., Chiang T.-Y., Lin P.-Y., Tsai T.-D., Chang Y.-C., Guo T.-F., Chen P., Wen T.-C., Hsu Y.-J. (2014). Nickel Oxide Electrode Interlayer in CH_3_NH_3_PbI_3_ Perovskite/PCBM Planar-Heterojunction Hybrid Solar Cells. Adv. Mater..

[30] Liu Y., Page Z.A., Russell T.P., Emrick T. (2015). Finely Tuned Polymer Interlayers Enhance Solar Cell Efficiency. Angew. Chem. Int. Ed..

[31] Yeo J.-S., Kang R., Lee S., Jeon Y.-J., Myoung N., Lee C.-L., Kim D.-Y., Yun J.-M., Seo Y.-H., Kim S.-S. (2015). Highly efficient and stable planar perovskite solar cells with reduced graphene oxide nanosheets as electrode interlayer. Nano Energy.

[32] Zhang H., Xue L., Han J., Fu Y.Q., Shen Y., Zhang Z.-G., Li Y., Wang M. (2016). New generation perovskite solar cells with solution-processed amino-substituted perylene diimide derivative as electron-transport layer. J. Mater. Chem. A.

[33] Perrin D.D., Armarego W.L.F., Perrin D.R. (1980). Purification of Laboratory Chemicals.

[34] Raptopoulou C.P., Sanakis Y., Psycharis V., Pissas M. (2013). Zig-zag [Mn^III^_4_] clusters from polydentate Schiff base ligands. Polyhedron.

[35] Romadina E.I., Komarov D.S., Stevenson K.J., Troshin P.A. (2021). New phenazine based anolyte material for high voltage organic redox flow batteries. Chem. Commun..

[36] Casey K.G., Quitevis E.L. (1988). Effect of solvent polarity on nonradiative processes in xanthene dyes: Rhodamine B in normal alcohols. J. Phys. Chem..

[37] CrysAlisPro (2022). Rigaku Oxford Diffraction.

[38] Sheldrick G.M. (2015). SHELXT—Integrated space-group and crystal-structure determination. Acta Cryst..

[39] Sheldrick G.M. (2015). Crystal structure refinement with SHELXL. Acta Cryst..

[40] Dolomanov O.V., Bourhis L.J., Gildea R.J., Howard J.A.K., Puschmann H. (2009). OLEX2: A complete structure solution, refinement and analysis program. J. Appl. Cryst..

[41] Sheldrick G.M. (2008). A short history of SHELX. Acta Cryst. Sect. A.

[42] Macrae C.F., Sovago I., Cottrell S.J., Galek P.T.A., McCabe P., Pidcock E., Platings M., Shields G.P., Stevens J.S., Towler M. (2020). Mercury 4.0: From visualization to analysis, design and prediction. J. Appl. Cryst..

[43] Ansari I.A., Sama F., Raizada M., Shahid M., Rajpoot R.K., Siddiqi Z.A. (2017). Synthesis and spectral characterization of 2-((2-hydroxybenzylidene) amino)-2-methylpropane-1,3-diol derived complexes: Molecular docking and antimicrobial studies. J. Mol. Struct..

[44] Dey M., Rao C.P., Saarenketo P.K., Rissanen K., Kolehmainen E., Guionneau P. (2003). Mn(IV) and Co(III)-complexes of -OH-rich ligands possessing O_2_N, O_3_N and O_4_N cores: Syntheses, characterization and crystal structures. Polyhedron.

[45] Barman T.R., Sutradhar M., Drew M.G.B., Rentschler E. (2013). 2-Amino-2-methyl-1,3-propanediol (ampdH_2_) as ligand backbone for the synthesis of cobalt complexes: Mononuclear Co(II), binuclear Co(II,III) and hexanuclear Co(II,III). Polyhedron.

[46] Liimatainen J., Lehtonen A., Sillanpaa R. (2000). *cis*-Dioxomolybdenum(VI) complexes with tridentate and tetradentate Schiff base ligands. Preparation, structures and inhibition of aerial oxidation of aldehydes. Polyhedron.

[47] Kato M., Nakajima K., Yoshikawa Y., Hirotsu M., Kojima M. (2000). Preparation and properties of dinuclear dioxomolybdenum(VI) complexes with ONO–ONO-type hexadentate Schiff base ligands. Inorg. Chim. Acta.

[48] Dey M., Rao C.P., Saarenketo P., Rissanen K., Kolehmainen E. (2002). Four-, Five- and Six-Coordinated Zn^II^ Complexes of OH-Containing Ligands: Syntheses, Structure and Reactivity. Eur. J. Inorg. Chem..

[49] Asgedom G., Sreedhara A., Kivikoski J., Valkonen J., Kolehmainen E., Rao C.P. (1996). Alkoxo Bound Monooxo- and Dioxovanadium(V) Complexes: Synthesis, Characterization, X-ray Crystal Structures, and Solution Reactivity Studies. Inorg. Chem..

[50] Rao C.P., Sreedhara A., Rao P.V., Lokanath N.K., Sridhar M.A., Prasad J.S., Rissanen K. (1999). Recognition of oxovanadium(V) species and its separation from other metal species through selective complexation by some acyclic ligands. Polyhedron.

[51] Krylova I.V., Saverina E.A., Rynin S.S., Lalov A.V., Minyaev M.E., Nikolaevskaya E.N., Syroeshkin M.A., Egorov M.P. (2020). Synthesis, characterization and redox properties of Ar–C=N→Ge←N=C–Ar containing system. Mendeleev Commun..

[52] Yearwood B., Parkin S., Atwood D.A. (2002). Synthesis and characterization of organotin Schiff base chelates. Inorg. Chim. Acta.

[53] Jastrzebski J.T.B.H., Van Koten G. (1993). Intramolecular Coordination in Organotin Chemistry. Adv. Organomet. Chem..

[54] Baul T.S.B., Addepalli M.R., Lyčka A., van Terwingen S., Guedes da Silva M.F.C. (2020). Synthesis and structural characterization of diorganotin(IV) complexes with heteroditopic pyridyl-ONO′-ligands. Inorg. Chim. Acta.

[55] Gock M., Wiedemann B., Dietz C., Bai C., Lutter M., Abeyawarathan V., Jurkschat K. (2013). Simplicity Meets Beauty. Trapping Molecular Dimethyltin Oxide in the Novel Organotinoxo Cluster [MeN(CH_2_CH_2_O)_2_SnMe_2_·Me_2_SnO]_3_. Organometallics.

[56] Rabiee N., Safarkhani M., Amini M.M. (2019). Investigating the structural chemistry of organotin(IV) compounds: Recent advances. Rev. Inorg. Chem..

[57] Broere D.L.J., Plessius R., van der Vlugt J.I. (2015). New avenues for ligand-mediated processes—Expanding metal reactivity by the use of redox-active catechol, o-aminophenol and o-phenylenediamine ligands. Chem. Soc. Rev..

[58] Hoijtink G.J. (1958). Oxidation potentials of conjugated hydrocarbons. Recueil des Travaux Chimiques des Pays-Bas.

[59] Berlman I. (1971). Handbook of Fluorescence Spectra of Aromatic Molecules.

[60] Fobbe H., Neumann W.P. (1986). Organozinnverbindungen: XXXII. Zur photochemischen reaktion von ketonen mit tetramethyl-1,2-bis(phenylthio)distannan. J. Organomet. Chem..

[61] Scherping K.H., Neumann W.P. (1982). Chemistry of heavy carbene analogs, R_2_M (M = Si, Ge, Sn). 7. A new and convenient source for stannylenes, R_2_Sn: 1,2-bis(phenylthio)tetramethyldistannane Me_2_(PhS)SnSn(SPh)Me_2_. Organometallics.

[62] Che Y.-X., Qi X.-N., Qu W.-J., Shi B.-B., Lin Q., Yao H., Zhang Y.-M., Wei T.-B. (2022). Synthetic strategies of phenazine derivatives: A review. J. Heterocycl. Chem..

[63] Mavrodi D.V., Blankenfeldt W., Thomashow L.S. (2006). Phenazine compounds in fluorescent Pseudomonas spp. biosynthesis and regulation. Annu. Rev. Phytopathol..

[64] Wei T.-B., Li W.-T., Li Q., Su J.-X., Qu W.-J., Lin Q., Yao H., Zhang Y.-M. (2016). A turn-on fluorescent chemosensor selectively detects cyanide in pure water and food sample. Tetrahedron Lett..

[65] Elgrishi N., Rountree K.J., McCarthy B.D., Rountree E.S., Eisenhart T.T., Dempsey J.L. (2018). A Practical Beginner’s Guide to Cyclic Voltammetry. J. Chem. Educ..

[66] Akbulatov A.F., Frolova L.A., Griffin M.P., Gearba I.R., Dolocan A., Vanden Bout D.A., Tsarev S., Katz E.A., Shestakov A.F., Stevenson K.J. (2017). Effect of Electron-Transport Material on Light-Induced Degradation of Inverted Planar Junction Perovskite Solar Cells. Adv. Energy Mater..

[67] Elnaggar M., Boldyreva A.G., Elshobaki M., Tsarev S.A., Fedotov Y.S., Yamilova O.R., Bredikhin S.I., Stevenson K.J., Aldoshin S.M., Troshin P.A. (2020). Decoupling Contributions of Charge-Transport Interlayers to Light-Induced Degradation of P-i-n Perovskite Solar Cells. Sol. RRL.

