# Supplementary material for: Sn and Ge Complexes with Redox-Active Ligands as Efficient Interfacial Membrane-like Buffer Layers for p-i-n Perovskite Solar Cells
Source: Membranes (Basel). 2023 Apr 17;13(4):439. doi: 10.3390/membranes13040439 (PMC10145979; doi:10.3390/membranes13040439)
Supplement: Supplementary file 1 [file membranes-13-00439-s001.zip › membranes-2313638-supplementary.pdf]

Supporting Information

Sn and Ge complexes with redox-active ligands as efficient interfacial membrane-like buffer layers for p-i-n perovskite solar cells

Azat F. Akbulatov[1], Anna Ya. Akyeva[2], Pavel G. Shangin[2], Nikita A. Emelianov[1], Irina V. Krylova[2], Mariya O. Markova[2,3], Liliya D. Labutskaya[2,4], Alexander V. Mumyatov[1], Egor I. Tuzharov[2], Dmitry A. Bunin[5], Lyubov A. Frolova[1], Mikhail P. Egorov[2], Mikhail A. Syroeshkin[2], and Pavel A. Troshin[1]

[1] Federal Research Center of Problems of Chemical Physics and Medicinal Chemistry, Russian Academy of Sciences, Academician Semenov ave. 1, Chernogolovka, Moscow Region, 142432 Russia

[2] N.D. Zelinsky Institute of Organic Chemistry, Russian Academy of Sciences, Moscow, 119991 Russia

[3] Dmitry Mendeleev University of Chemical Technology of Russia, Moscow, Russia

[4] Sechenov First Moscow State Medical University, Moscow, Russia

[5] A.N. Frumkin Institute of Physical Chemistry and Electrochemistry, Russian Academy of Sciences, Moscow, Russia

Correspondence: troshin2003@inbox.ru or troshin@icp.ac.ru

Contents

Table S1. Crystal data, data collection and structure refinement details refinement for **1**........ 4
Figure S1. The molecular structure of **1** (p = 50%)........ 5
Table S2. Selected bond lengths [Å] for **1**........ 5
Table S3. Selected bond angles [°] for **1**........ 6
Table S4. Hydrogen bonds for **1** [Å and °]........ 6
Figure S2. Hydrogen bonding in **1**........ 6
Figure S3. ^{1}H spectrum of compound **1**........ 7
Figure S4. ^{13}C NMR spectrum of compound **1**........ 7
Figure S5. HRMS spectra of compound **1**........ 8
Figure S6. ^{1}H NMR spectrum of compound **2**........ 9
Figure S7. ^{13}C NMR spectrum of compound **2**........ 9
Figure S8. HSQC NMR spectrum of compound **2**........ 10
Figure S9. HRMS spectra of **2**........ 11
Figure S10. ^{1}H NMR spectrum of compound **3**........ 12
Figure S11. ^{13}C NMR spectrum of compound **3**........ 12
Figure S12. HSQC NMR spectrum of compound **3**........ 13
Figure S13. HRMS spectra of **3**........ 14
Figure S14. ^{1}H NMR spectrum of compound **4**........ 15
Figure S15. HRMS spectrum of **4**........ 16
Figure S16. ESI-HRMS spectra (negative ion mode, MeOH) of the germanium dianion (*z* = 2) of **5**........ 17
Figure S17. ^{1}H NMR spectrum of compound **5**........ 17

Figure S18. HRMS spectrum of **5**………………………………………………………………………………… 18
Figure S19. CV curves of oxidation and reduction of **2** in a 0.1 M Bu_4NBF_4/DMF supporting electrolyte on a glassy carbon disc electrode at a potential scan rate of 100 mV s^{-1}. Absorbance and fluorescence spectra of **2** in DMF…………………………………………… 19
Figure S20. CV curves of oxidation and reduction of **3** in a 0.1 M Bu_4NBF_4/DMF supporting electrolyte on a glassy carbon disc electrode at a potential scan rate of 100 mV s^{-1}. Absorbance and fluorescence spectra of **3** in DMF. ………………………………………… 19
Figure S21. CV curves of oxidation and reduction of **5** in a 0.1 M Bu_4NBF_4/DMF supporting electrolyte on a glassy carbon disc electrode at a potential scan rate of 100 mV s^{-1}. Absorbance and fluorescence spectra of **5** in DMF…………………………………………… 20
Figure S22. Thermal gravimetry profiles of compounds **1** (a), **2** (b), **3** (c), **4** (d), **5** (e) and **6** (f). 21
Table S5. Modification of surface properties of $PC_{61}BM$ by interlayers **1-6** 21
Figure S23. *J-V* curves and EQE spectra of perovskite solar cells with different concentrations of compound **1**………………………………………………………………………………… 22
Table S6. Photovoltaic parameters of perovskite solar cells using compound **1** as interlayer………………………………………………………………………………………………….. 23
Figure S24. V_{OC}, J_{SC}, FF and PCE of PSCs as a function of concentration of **2**. *J*-V curves and EQE of the best devices………………………………………………………………………… 23
Table S7. Photovoltaic parameters of best solar cells with using of **2** as interlayer………… 24
Figure S25. V_{OC}, J_{SC}, FF and PCE of PSCs as a function of concentration of **3**, I-V curves and EQE of the best devices………………………………………………………………………… 24
Table S8. Photovoltaic parameters of best solar cells with using of **3** as interlayer………… 25
Figure S26. V_{OC}, J_{SC}, FF and PCE of PSCs as a function of concentration of **4**, I-V curves and EQE of the best devices………………………………………………………………………… 25
Table S9. Photovoltaic parameters of best solar cells with using of **4** as interlayer………… 26
Figure S27. V_{OC}, J_{SC}, FF and PCE of PSCs as a function of concentration of **5**, I-V curves and EQE of the best devices………………………………………………………………………… 26
Table S10. Photovoltaic parameters of best solar cells with using of **5** as interlayer………… 27
Figure S28. V_{OC}, J_{SC}, FF and PCE of PSCs as a function of concentration of **6**, I-V curves 27
and EQE of the best devices………………………………………………………………………… 28
Table S11. Photovoltaic parameters of best solar cells with using of **6** as interlayer………. 28
Figure S29. The evolution of the normalized open-circuit voltage (a), short-circuit current (b), fill factor (c) and power conversion efficiency (d) of perovskite solar cells using bare $PC_{61}BM$ and its combination with compound 1 as ETL materials. 29
Figure S30. ATR FTIR spectra of $MAPbI_3$, $PC_{61}BM$ and **1**……………………………………………….. 29
Figure S31. ATR FTIR spectra of $MAPbI_3$, $PC_{61}BM$ and **2**………………………………………………..
Figure S32. AFM topography of ITO/PTA/$MAPbI_3$/$PC_{61}BM$/**2** film; mappings of
ITO/PTA/$MAPbI_3$/$PC_{61}BM$/**2** topography at frequencies of 962 cm^{-1}, 1738 cm^{-1}, and 30
1002 cm^{-1}, which are characteristic for $MAPbI_3$, $PC_{61}BM$, and **2**, respectively… 30
Figure S33. ATR FTIR spectra of $MAPbI_3$, $PC_{61}BM$ and **3**………………………………………………..
Figure S34. AFM topography of ITO/PTA/$MAPbI_3$/$PC_{61}BM$/**3** film; mappings of
ITO/PTA/$MAPbI_3$/$PC_{61}BM$/**3** topography at frequencies of 1249 cm^{-1}, 1738 cm^{-1} and 31
1519 cm^{-1}, which are characteristic for $MAPbI_3$, $PC_{61}BM$, and **3**, respectively 31
Figure S35. ATR FTIR spectra of $MAPbI_3$, $PC_{61}BM$ and **4**………………………………………………..
Figure S36. AFM topography of ITO/PTA/$MAPbI_3$/$PC_{61}BM$/**4** film; mappings of
ITO/PTA/$MAPbI_3$/$PC_{61}BM$/**4** topography at frequencies of 962 cm^{-1}, 1738 cm^{-1} and 1146 32
cm^{-1}, which are characteristic for **4**, $PC_{61}BM$, and $MAPbI_3$, respectively………………… 32

Figure S37. ATR FTIR spectra of $MAPbI_3$, $PC_{61}BM$ and **5**………………………………………………….
Figure S38. AFM topography of ITO/PTA/$MAPbI_3$/$PC_{61}BM$/**5** film; mappings of ITO/PTA/$MAPbI_3$/$PC_{61}BM$/**5** topography at frequencies of 962 cm^{-1}, 1738 cm^{-1}, and 1199 cm^{-1}, which are characteristic for **5**, $PC_{61}BM$, and $MAPbI_3$, respectively…………… 33
Figure S39. ATR FTIR spectra of $MAPbI_3$, $PC_{61}BM$ and **6**………………………………………………….
Figure S40. AFM topography of ITO/PTA/$MAPbI_3$/$PC_{61}BM$/**6** film; mappings of ITO/PTA/$MAPbI_3$/$PC_{61}BM$/**6** topography at frequencies of 962 cm^{-1}, 1738 cm^{-1}, and 1586 cm^{-1}, which are characteristic for **6**, $PC_{61}BM$, and $MAPbI_3$, respectively……………

Table S1. Crystal data, data collection and structure refinement details refinement for **1**.

Empirical formula	C30 H46 N2 O6 Sn2	
Formula weight	768.07	
Temperature	100.15 K	
Wavelength	0.71073 Å	
Crystal system	Monoclinic	
Space group	$P2_1/c$	
Unit cell dimensions	a = 8.34430(10) Å	α = 90°.
	b = 12.85750(10) Å	β = 101.7940(10)°.
	c = 14.5864(2) Å	γ = 90°.
Volume	1531.89(3) $Å^3$	
Z	2	
Density (calculated)	1.665 g/cm^3	
Absorption coefficient	1.675 mm^{-1}	
F(000)	776	
Crystal size	0.12 x 0.04 x 0.03 mm^3	
Theta range for data collection	2.131 to 35.833°.	
Index ranges	-13<=h<=13, -20<=k<=19, -22<=l<=23	
Reflections collected	59080	
Independent reflections	6721 [R(int) = 0.0454]	
Observed reflections	5974	
Completeness to θ_{full} = 25.242°	1.000	
Max. and min. transmission	1.00000 and 0.55356	
Data / restraints / parameters	6721 / 4 / 201	
Goodness-of-fit on F^2	1.038	
Final R indices [I>2σ(I)]	R1 = 0.0234, wR2 = 0.0547	
R indices (all data)	R1 = 0.0293, wR2 = 0.0575	
Largest diff. peak and hole	1.509 and -0.991 e.$Å^{-3}$	

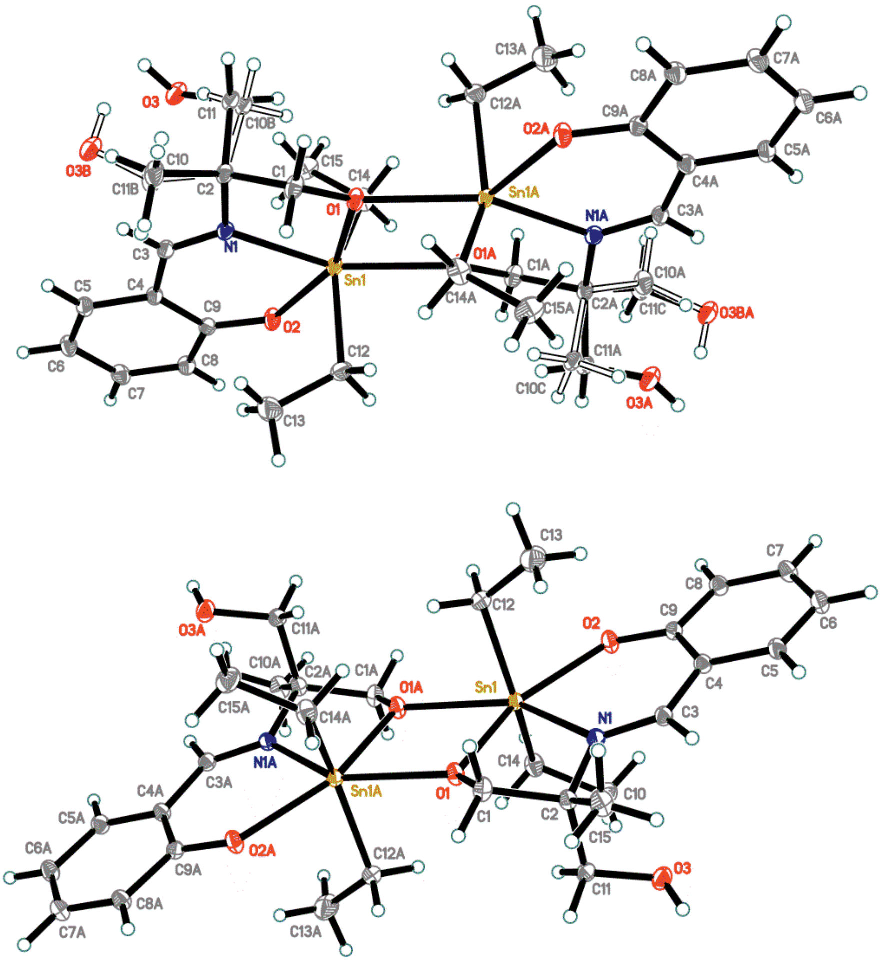

Figure S1. The molecular structure of **1** (p = 50%). A minor component of the OH disorder is shown in open solid lines; the disorder ratio is 0.909(3):0.091(3) (top). The disorder is omitted (bottom).

Table S2. Selected bond lengths [Å] for **1.**

Sn(1)-O(1)	2.0992(10)	N(1)-C(3)	1.2942(16)	O(3A)-C(11A)	1.412(3)
Sn(1)-O(1)#1	2.4090(10)	C(1)-C(2)	1.5358(19)	C(11A)-C(2)	1.545(2)
Sn(1)-O(2)	2.2658(10)	C(4)-C(3)	1.4431(18)	C(10A)-C(2)	1.528(2)
Sn(1)-N(1)	2.2369(11)	C(4)-C(9)	1.4242(19)	O(3B)-C(11B)	1.412(4)
Sn(1)-C(12)	2.1298(14)	C(4)-C(5)	1.4146(18)	C(11B)-C(2)	1.545(4)
Sn(1)-C(14)	2.1365(14)	C(6)-C(5)	1.3781(19)	C(10B)-C(2)	1.528(4)
O(2)-C(9)	1.3085(16)	C(6)-C(7)	1.400(2)	C(12)-C(13)	1.523(2)
O(1)-C(1)	1.4078(16)	C(8)-C(7)	1.3791(19)	C(14)-C(15)	1.528(2)
N(1)-C(2)	1.4850(17)	C(8)-C(9)	1.4175(18)		

Symmetry transformations used to generate equivalent atoms: #1 -x+1,-y,-z+1

Table S3. Selected bond angles [°] for **1.**

O(2)-Sn(1)-O(1)#1	133.29(3)	C(14)-Sn(1)-O(2)	84.83(5)
O(1)-Sn(1)-O(2)	157.64(4)	C(14)-Sn(1)-O(1)#1	83.85(5)
O(1)-Sn(1)-O(1)#1	69.07(4)	C(14)-Sn(1)-N(1)	105.92(5)
O(1)-Sn(1)-N(1)	76.33(4)	C(9)-O(2)-Sn(1)	132.51(8)
O(1)-Sn(1)-C(12)	101.62(5)	Sn(1)-O(1)-Sn(1)#1	110.93(4)
O(1)-Sn(1)-C(14)	100.17(5)	C(1)-O(1)-Sn(1)	115.20(8)
N(1)-Sn(1)-O(2)	81.34(4)	C(1)-O(1)-Sn(1)#1	128.70(8)
N(1)-Sn(1)-O(1)#1	145.23(4)	C(3)-N(1)-Sn(1)	128.28(9)
C(12)-Sn(1)-O(2)	84.13(5)	C(2)-N(1)-Sn(1)	113.04(8)
C(12)-Sn(1)-O(1)#1	81.73(5)	C(13)-C(12)-Sn(1)	115.66(10)
C(12)-Sn(1)-N(1)	102.52(5)	C(15)-C(14)-Sn(1)	115.35(10)
C(12)-Sn(1)-C(14)	147.46(6)		

Symmetry transformations used to generate equivalent atoms: #1 -x+1,-y,-z+1

Table S4. Hydrogen bonds for **1** [Å and °].

D-H...A	d(D-H)	d(H...A)	d(D...A)	<(DHA)
O(3B)-H(3B)...O(2)#2	0.83	2.35	3.036(12)	140.0
O(3A)-H(3A)...O(2)#2	0.83(3)	1.97(3)	2.7712(16)	161(3)

Symmetry transformations used to generate equivalent atoms: #1 -x+1, -y, -z+1 ; #2 x, -y+1/2, z+1/2

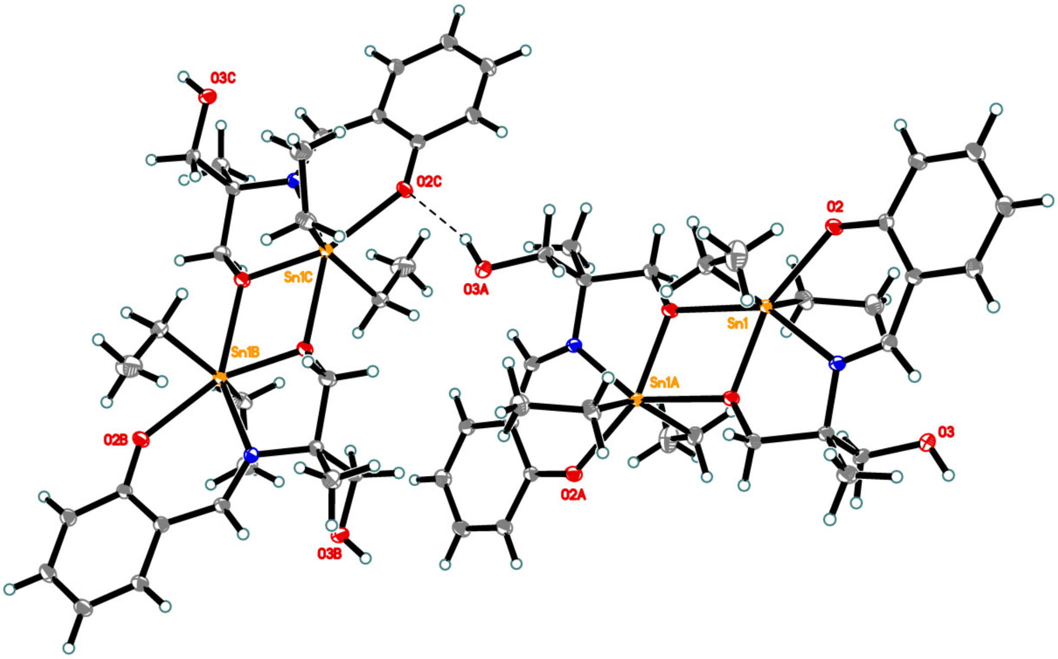

Figure S2. Hydrogen bonding in **1**. The OH disorder is omitted.

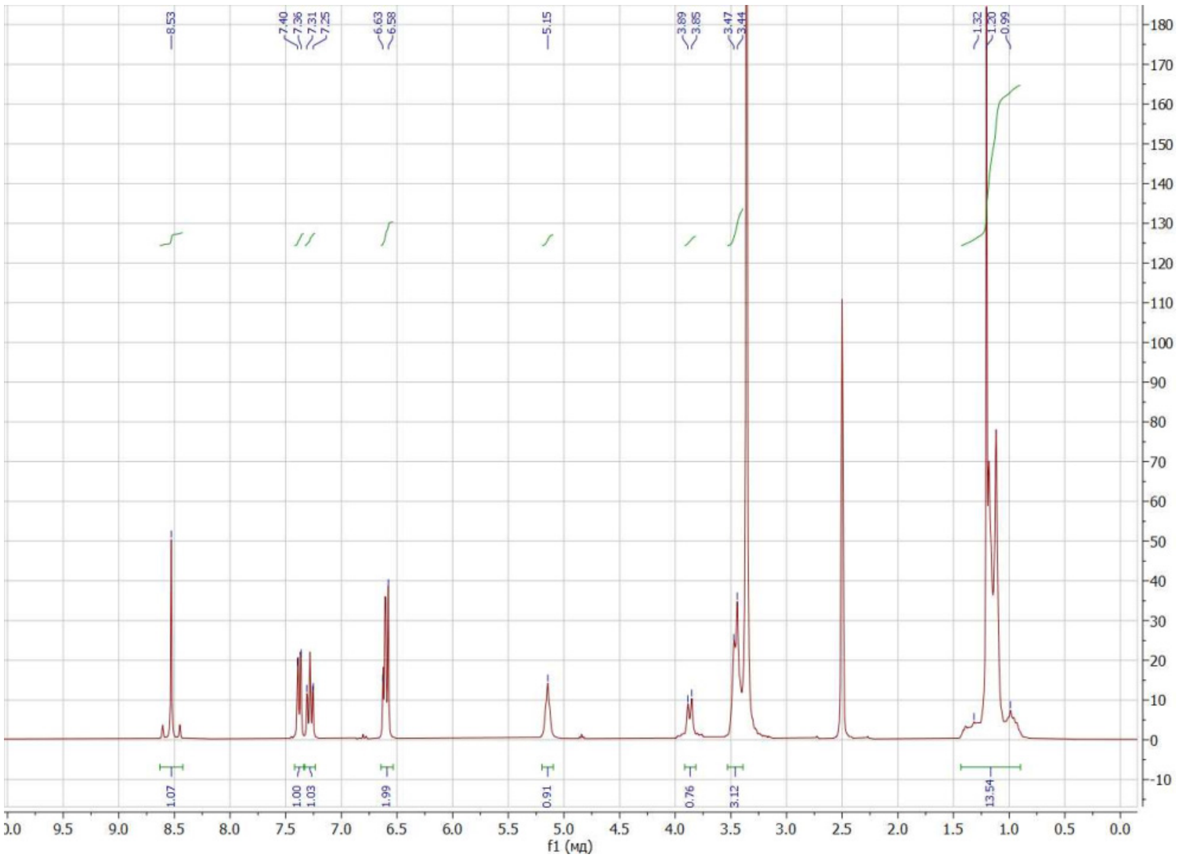

Figure S3. ^{1}H spectrum of compound **1**.

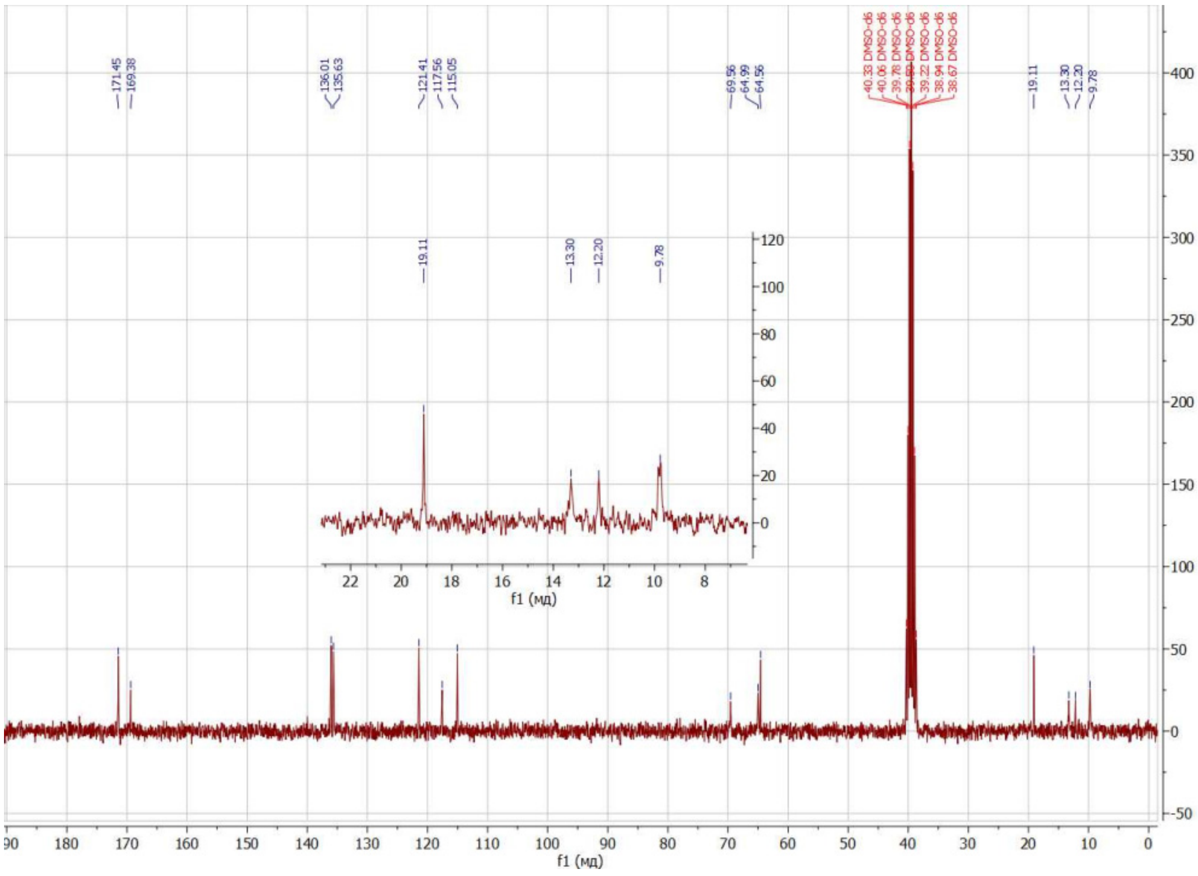

Figure S4. ^{13}C NMR spectrum of compound **1**.

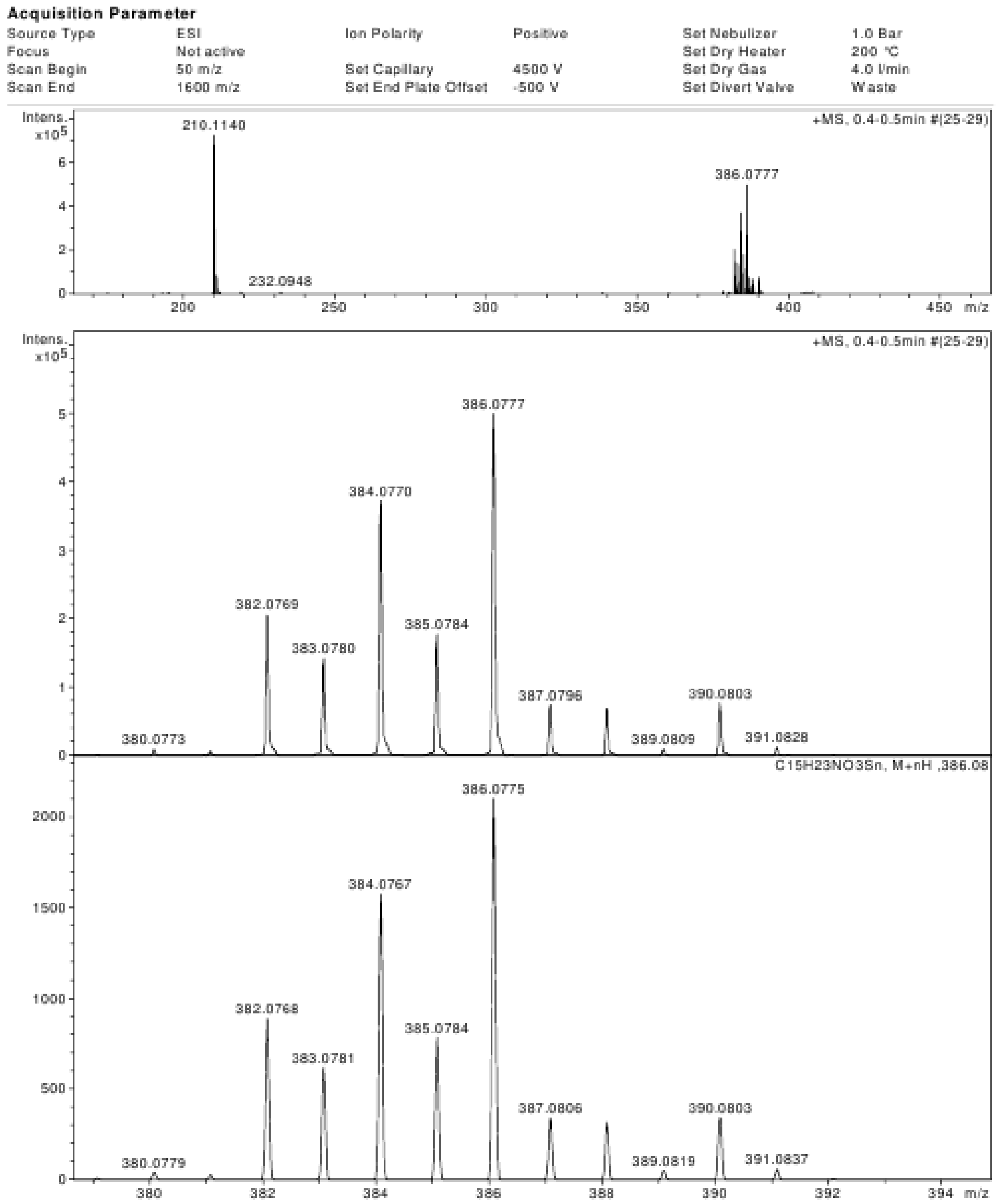

Figure S5. HRMS spectra of compound **1.**

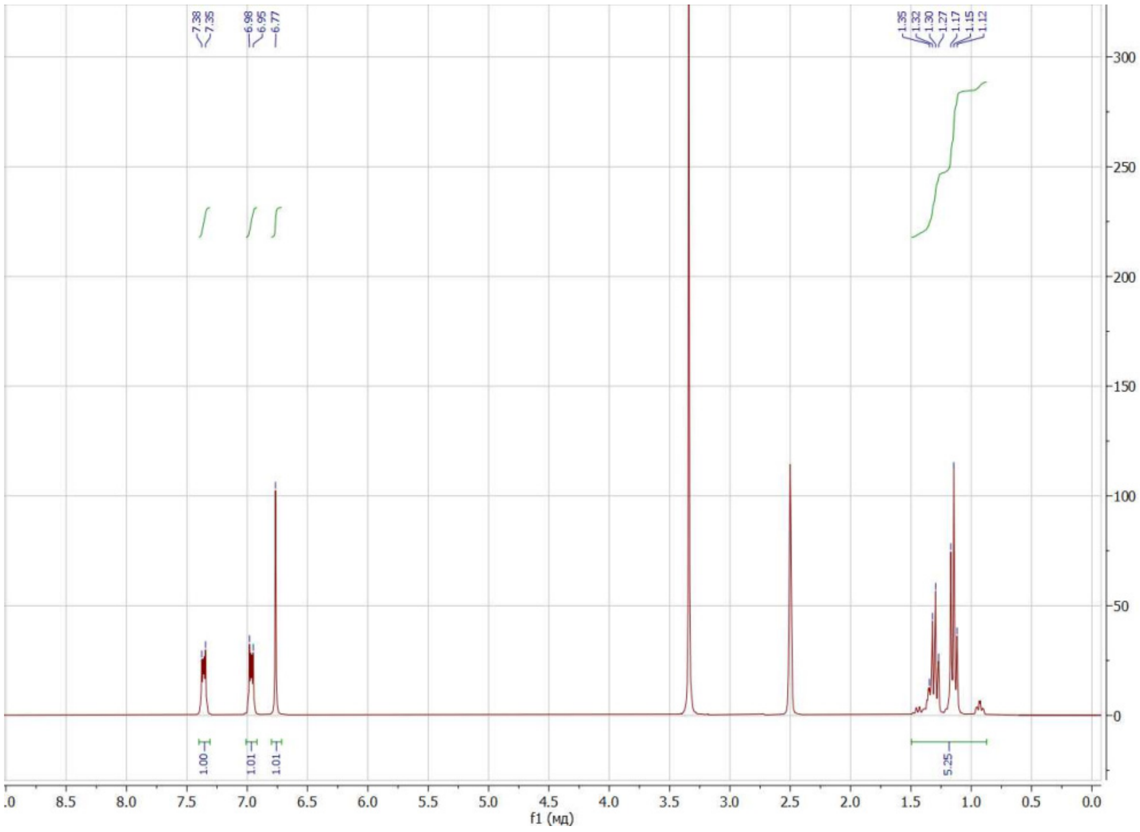

Figure S6. ^{1}H NMR spectrum of compound **2**.

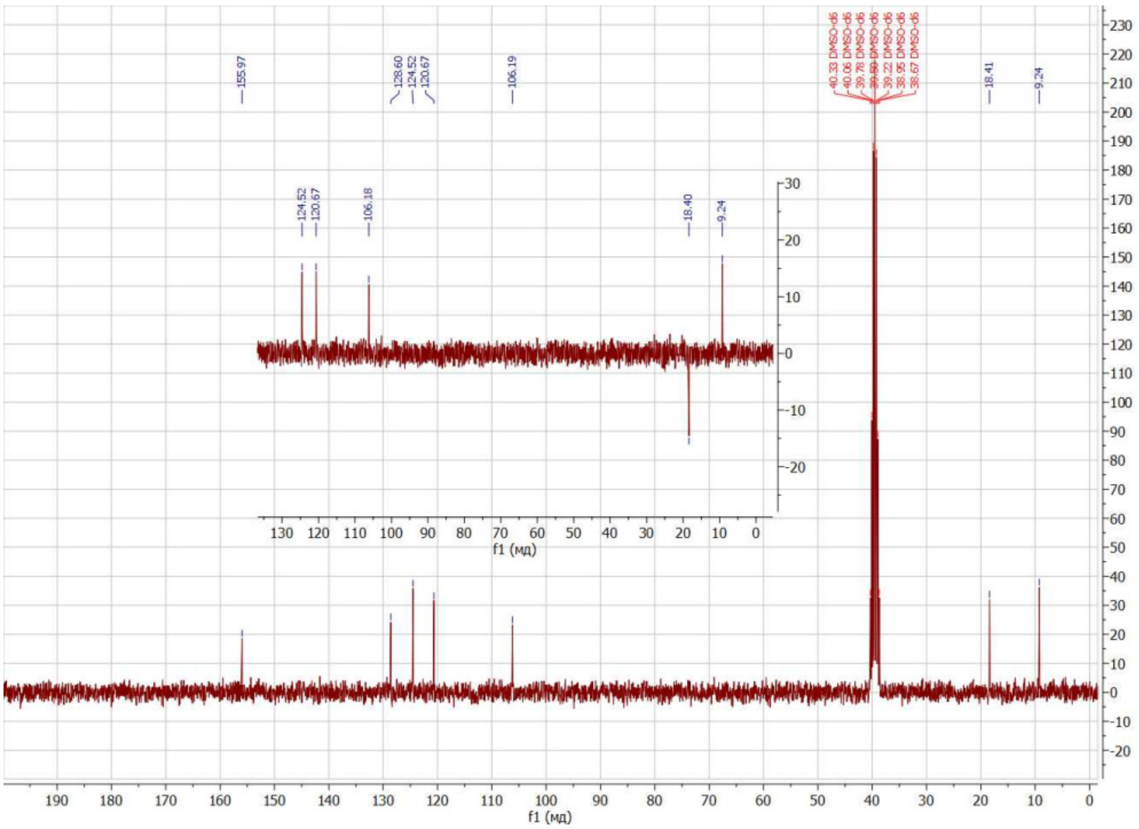

Figure S7. ^{13}C NMR spectrum of compound **2**.

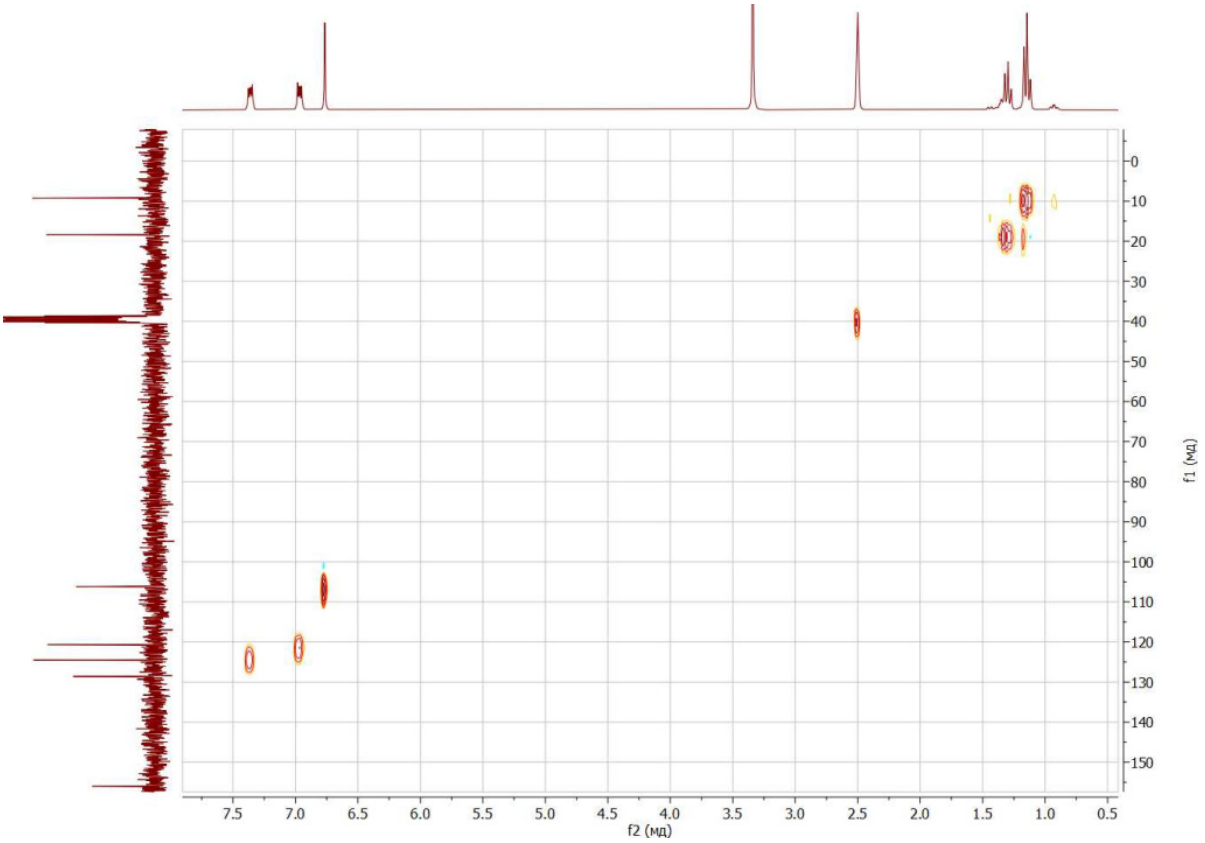

Figure S8. HSQC NMR spectrum of compound **2**.

Acquisition Parameter

Source Type	ESI	Ion Polarity	Positive	Set Nebulizer	1.0 Bar
Focus	Not active			Set Dry Heater	200 °C
Scan Begin	50 m/z	Set Capillary	4500 V	Set Dry Gas	4.0 l/min
Scan End	1600 m/z	Set End Plate Offset	-500 V	Set Divert Valve	Waste

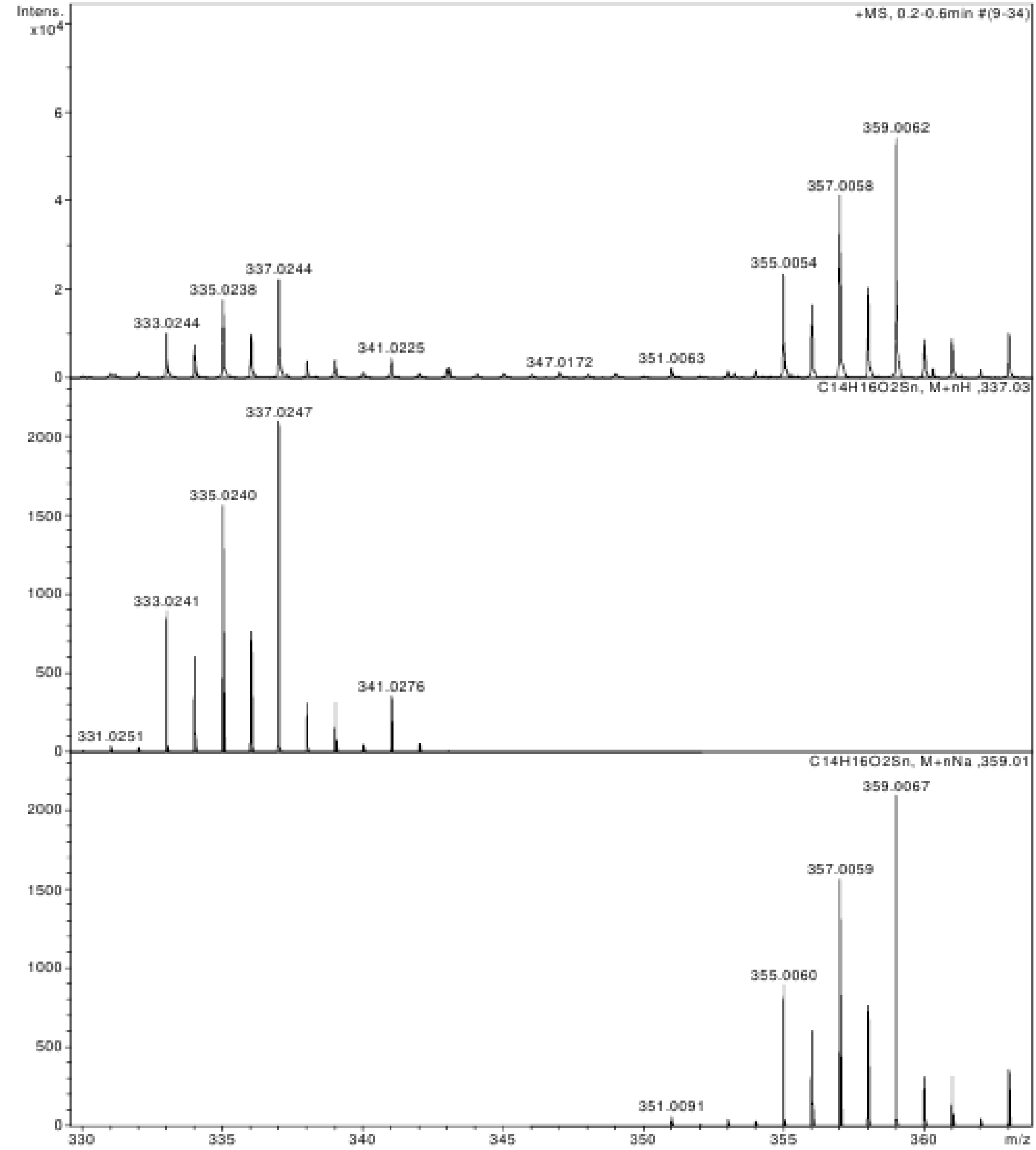

Figure S9. HRMS spectra of **2**.

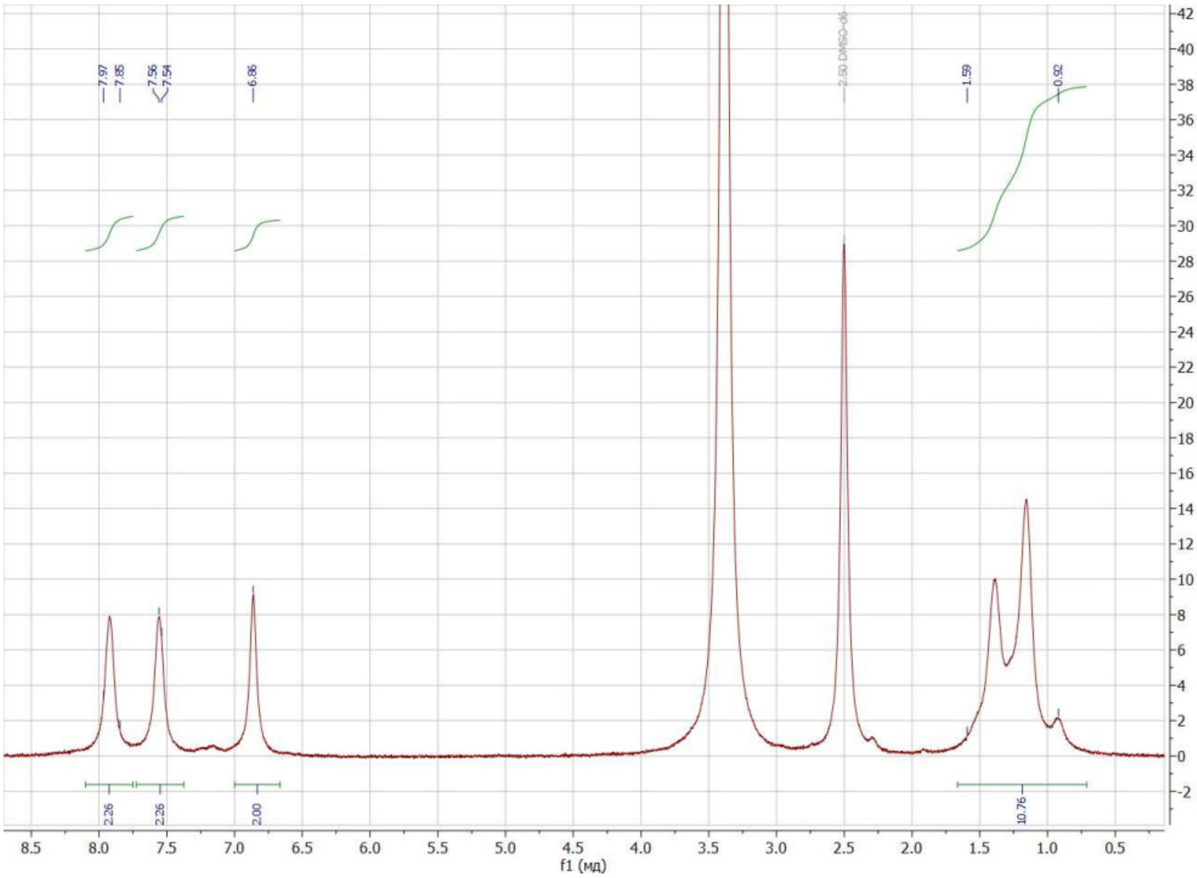

Figure S10. ^{1}H NMR spectrum of compound **3**.

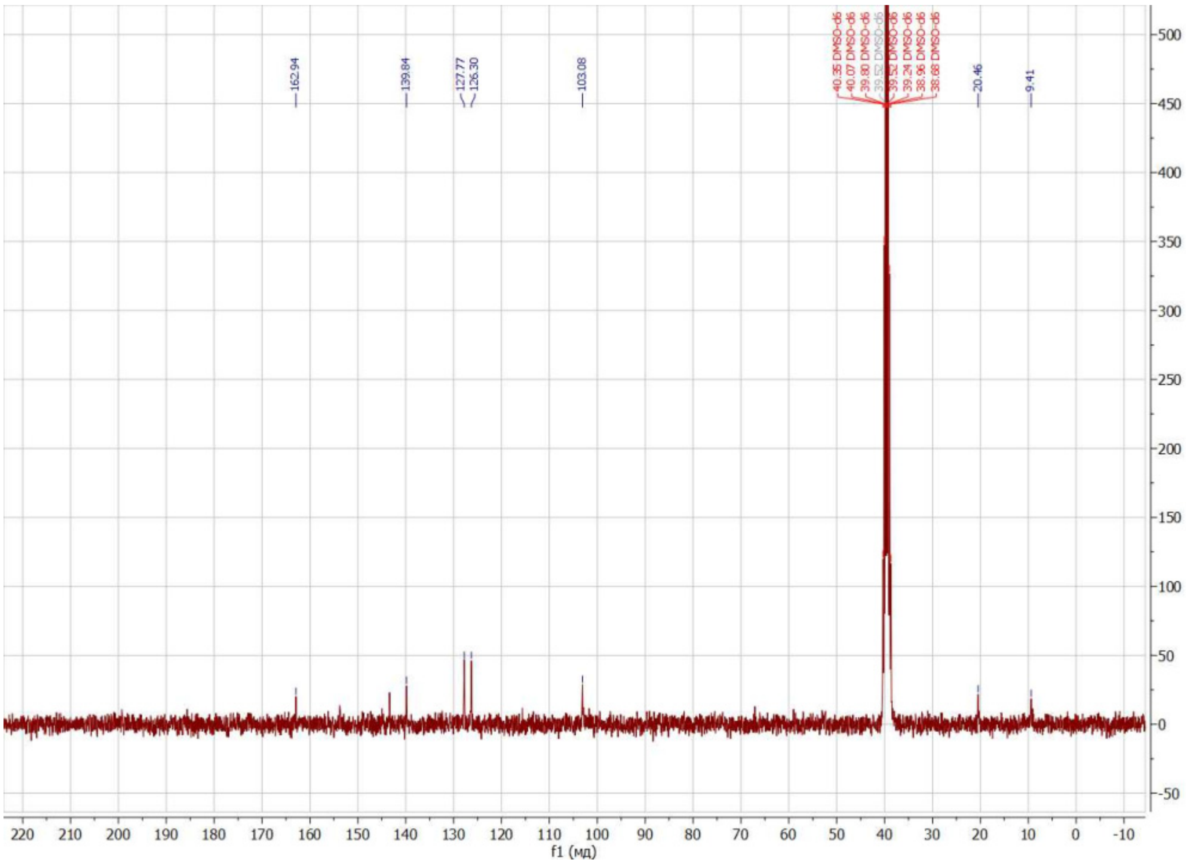

Figure S11. ^{13}C NMR spectrum of compound **3**.

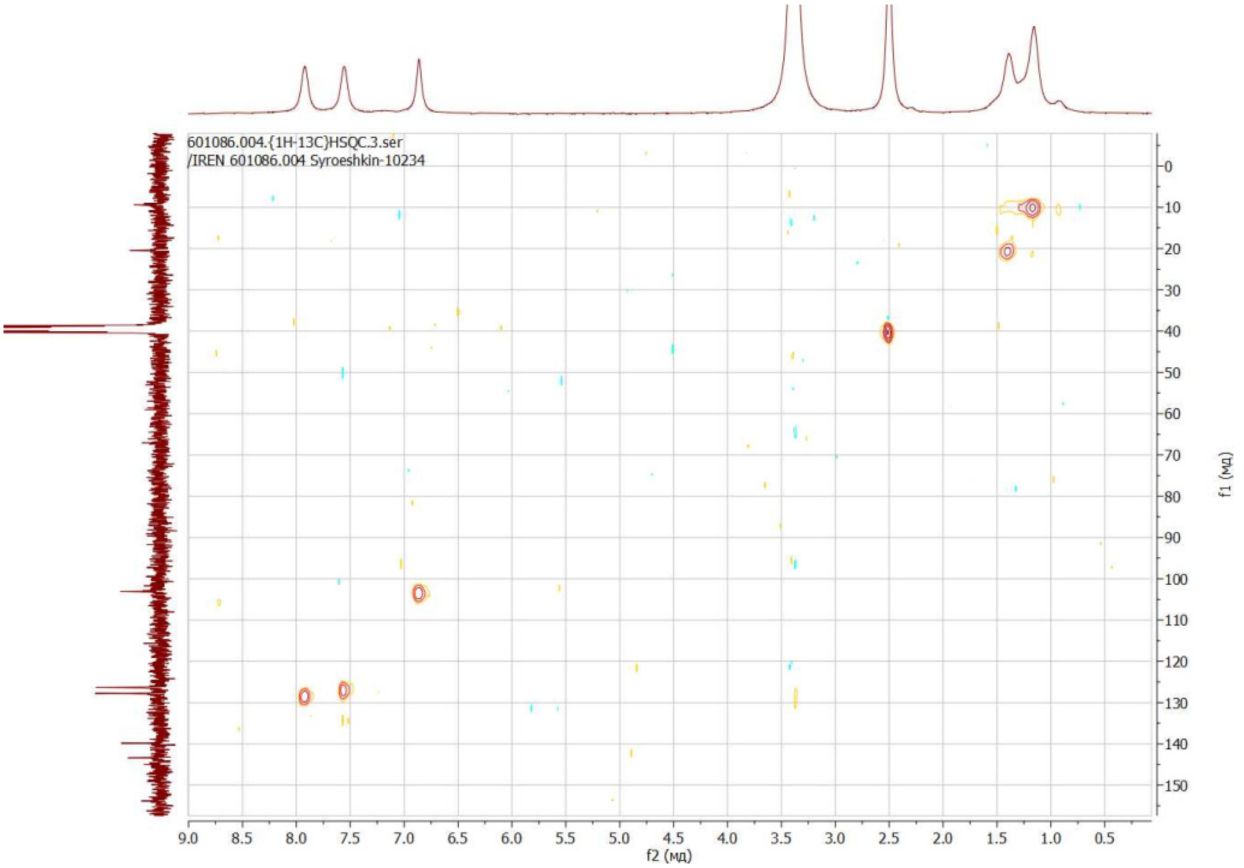

Figure S12. HSQC NMR spectrum of compound **3**.

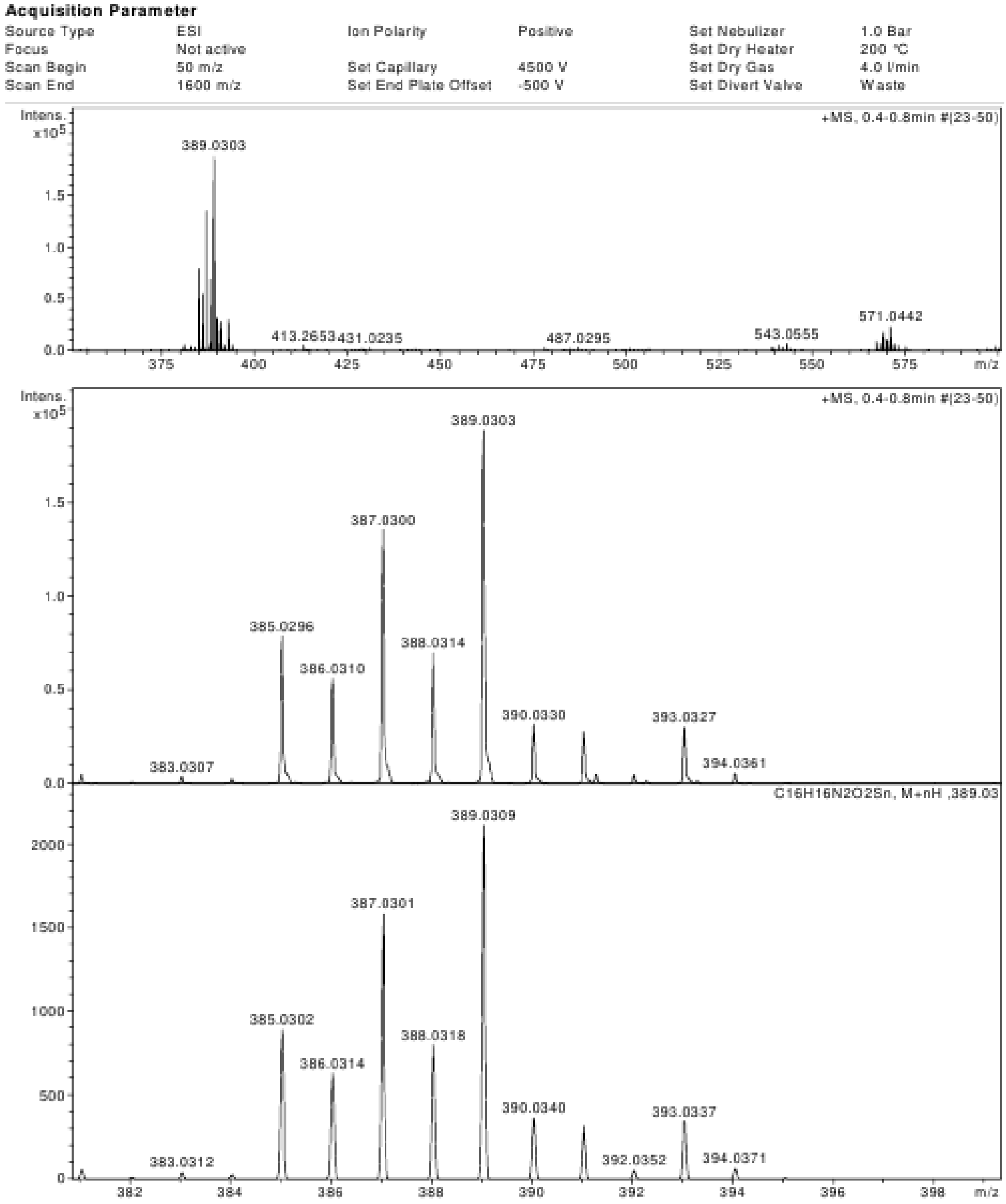

Figure S13. HRMS spectra of **3**.

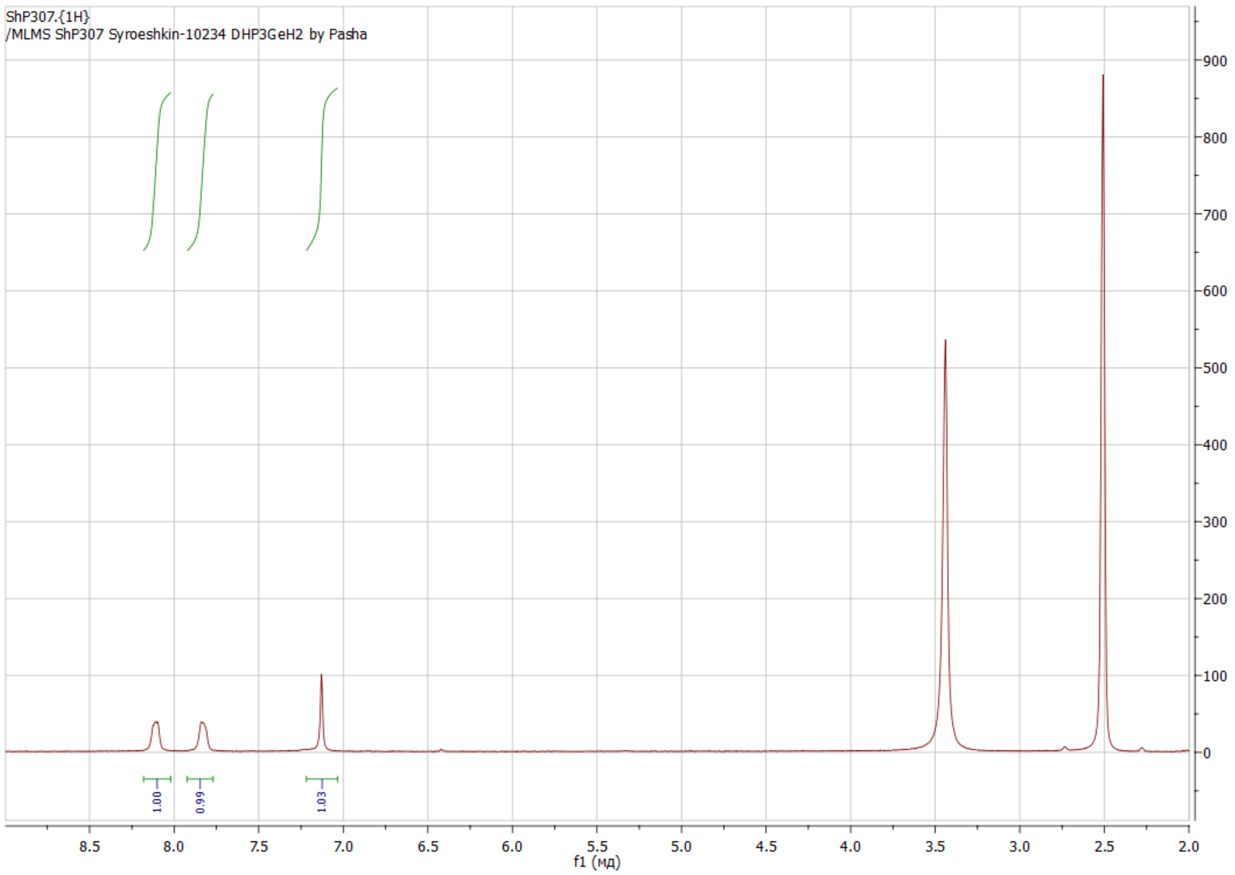

Figure S14. ^{1}H NMR spectrum of compound **4**.

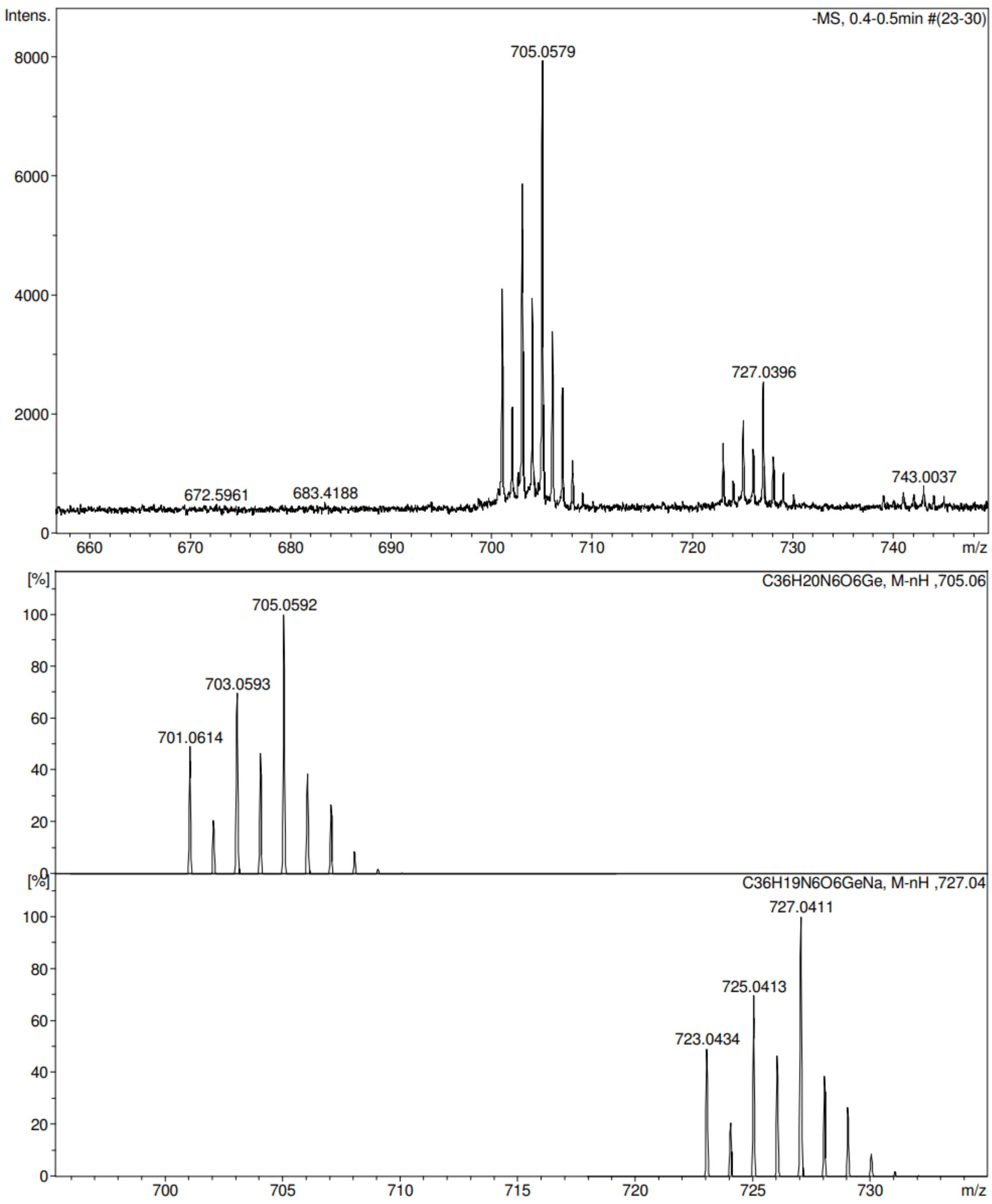

Figure S15. HRMS spectrum of **4**.

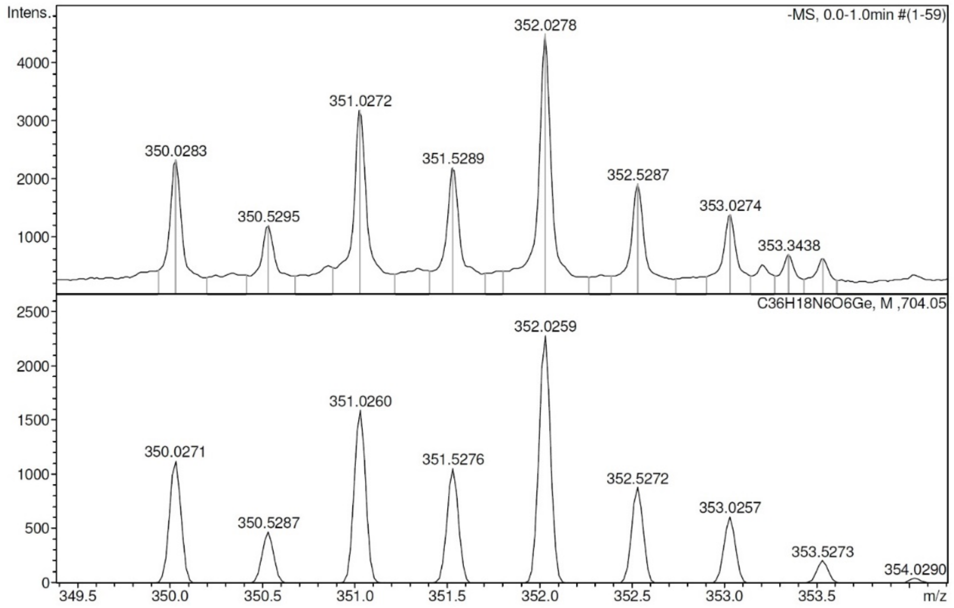

Figure S16. ESI-HRMS spectra (negative ion mode, MeOH) of the germanium dianion (z = 2) of **5**.

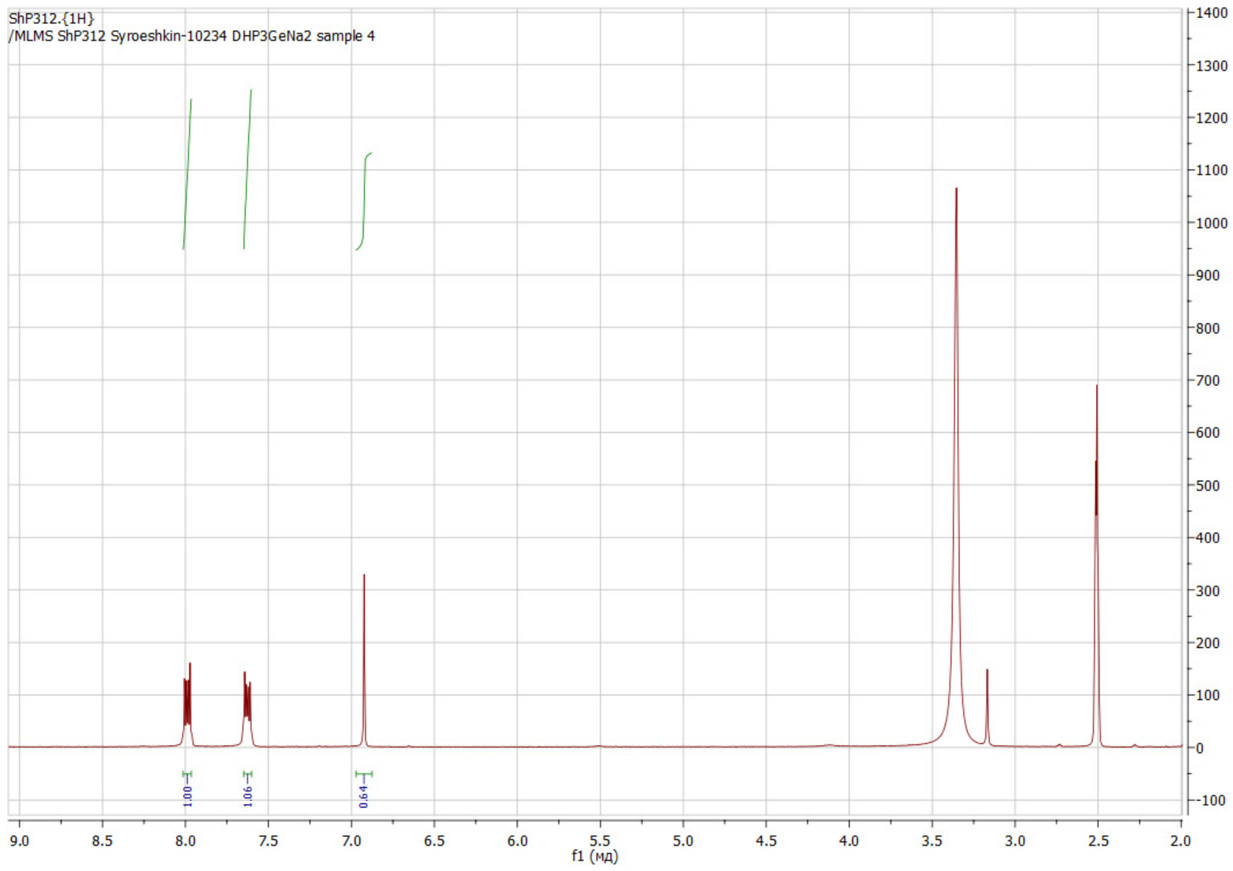

Figure S17. ^{1}H NMR spectrum of compound **5**.

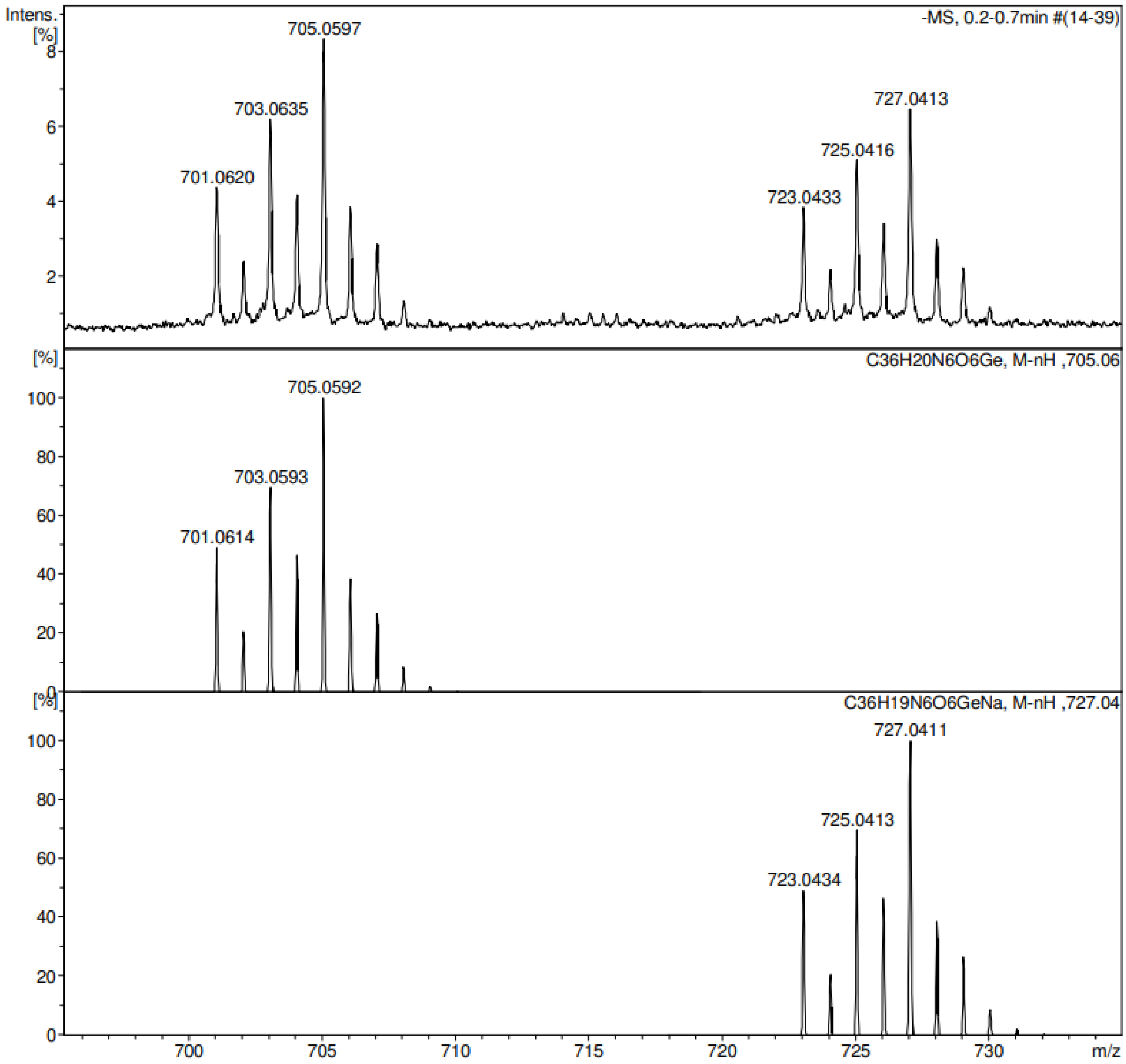

Figure S18. HRMS spectrum of **5**.

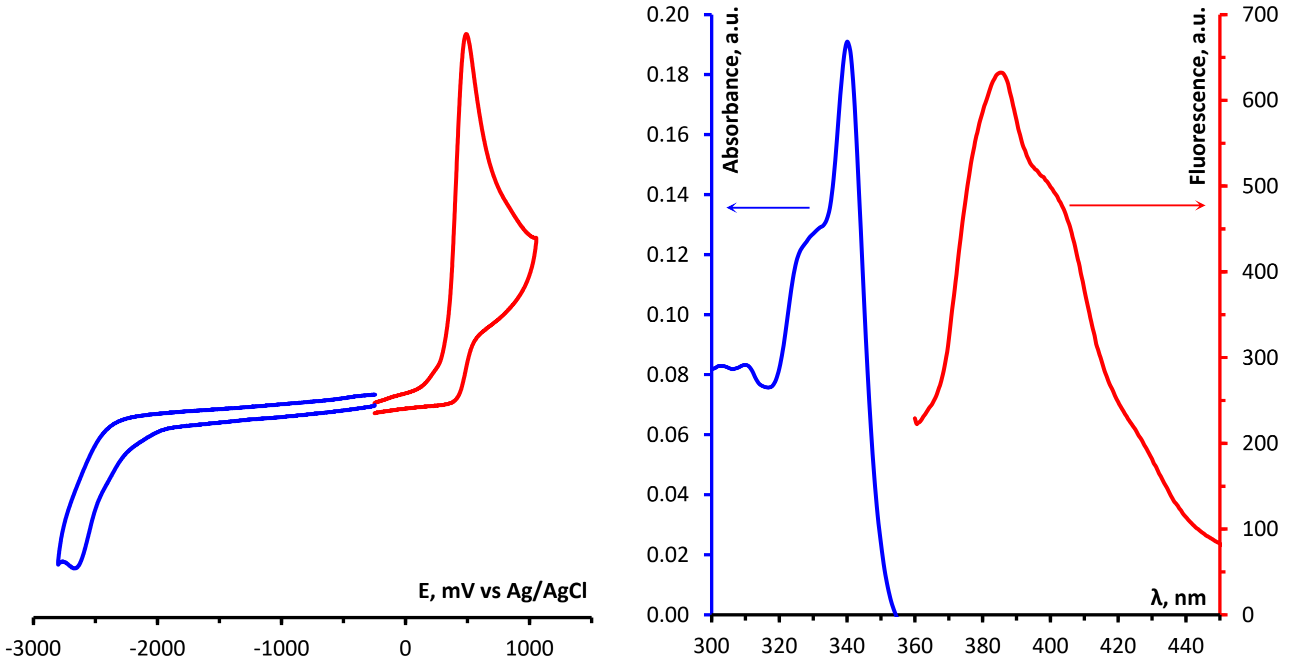

Figure S19. (*left*) CV curves of oxidation (*red*) and reduction (*blue*) of **2** (C = 3 mM) in a 0.1 M Bu_4NBF_4/DMF supporting electrolyte on a glassy carbon disc electrode at a potential scan rate of 100 mV s^{-1}. (*right*) Absorbance and fluorescence spectra of **2** in DMF (C = 0.1 mM).

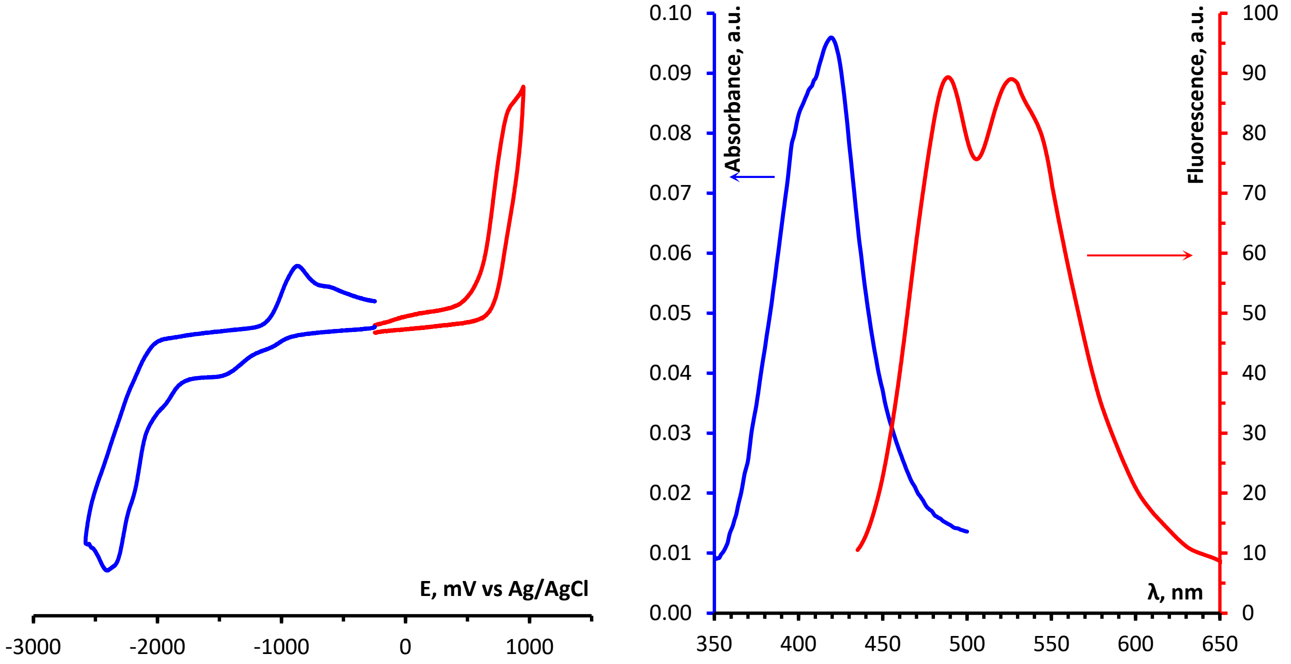

Figure S20. (*left*) CV curves of oxidation (*red*) and reduction (*blue*) of **3** (C = 3 mM) in a 0.1 M Bu_4NBF_4/DMF supporting electrolyte on a glassy carbon disc electrode at a potential scan rate of 100 mV s^{-1}. (*right*) Absorbance and fluorescence spectra of **3** in DMF (C = 0.1 mM).

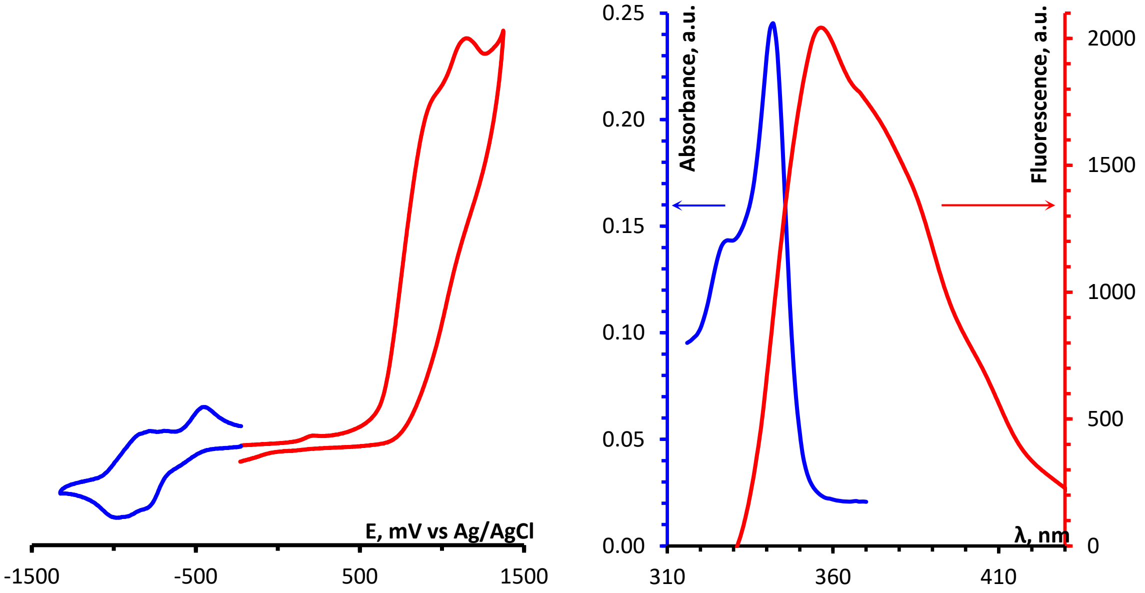

Figure S21. (*left*) CV curves of oxidation (*red*) and reduction (*blue*) of **5** (C = 3 mM) in a 0.1 M Bu_4NBF_4/DMF supporting electrolyte on a glassy carbon disc electrode at a potential scan rate of 100 mV s^{-1}. (*right*) Absorbance and fluorescence spectra of **5** in DMF.

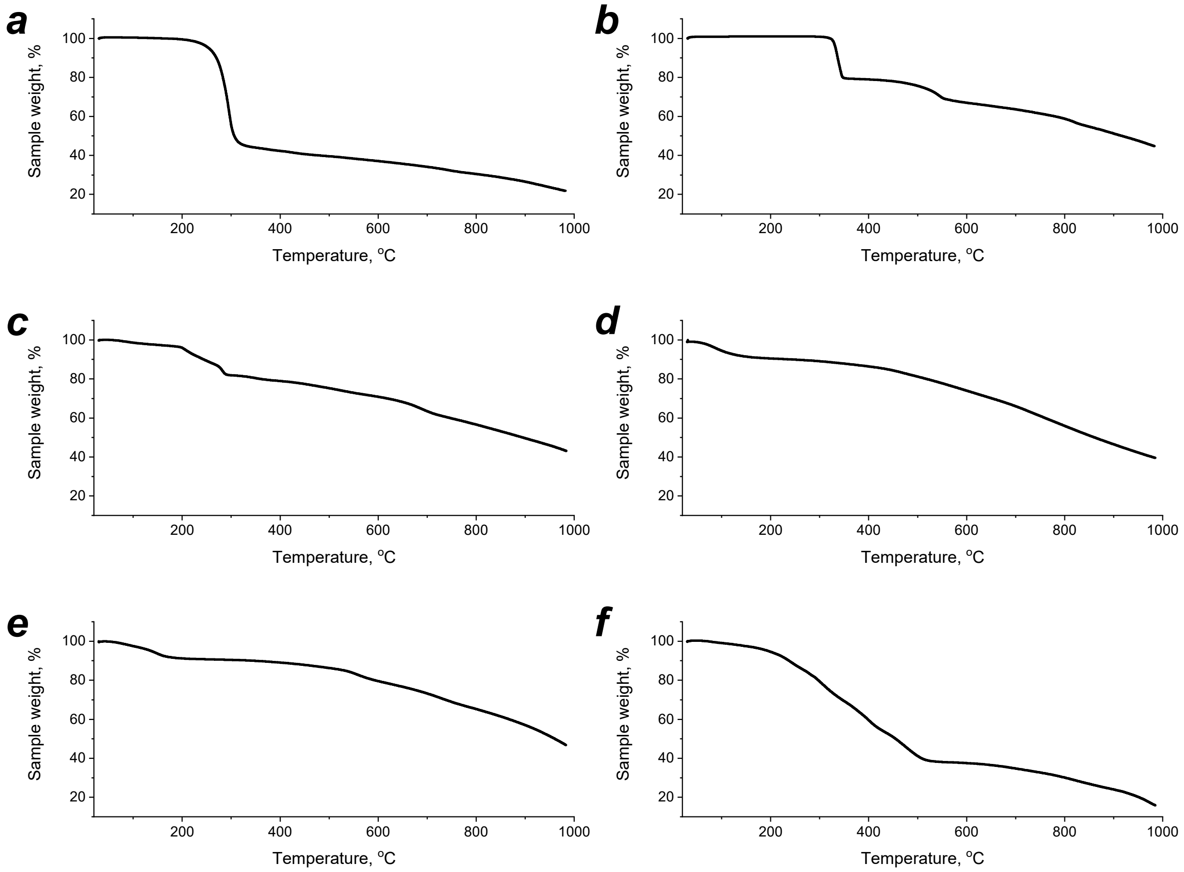

Figure S22. Thermal gravimetry profiles of compounds **1** (a), **2** (b), **3** (c), **4** (d), **5** (e) and **6** (f).

Table S5. Modification of surface properties of $PC_{61}BM$ by interlayers **1-6**

**Sample**	**Surface free energy, mN m^{-1}**	**Dispersive part, mN m^{-1}**	**Polar part, mN m^{-1}**	**Average water contact angle, deg.**	**Average CH_2I_2 contact angle, deg.**
$PC_{61}BM$/**1**	54.6±1.4	48.3±0.7	6.3±0.7	67.4±1.8	18.1±2.5
$PC_{61}BM$/**2**	49.2±2.1	47.1±1.8	2.1±0.4	80.6±1.1	22.1±2.5
$PC_{61}BM$/**3**	51.7±1.0	46.9±0.4	4.8±0.6	71.9±1.5	22.9±1.2
$PC_{61}BM$/**4**	50.4±0.7	46.4±0.2	4.0±0.5	74.6±1.3	24.4±0.6
$PC_{61}BM$/**5**	68.3±2.0	47.7±0.5	21.0±1.5	38.2±2.9	20.2±1.7
$PC_{61}BM$/**6**	52.8±1.6	47.7±1.1	5.2±0.6	70.6±1.4	20.4±3.5
$PC_{61}BM$	48.0±0.8	47.7±0.7	0.2±0.1	91.1±1.1	20.2±2.2

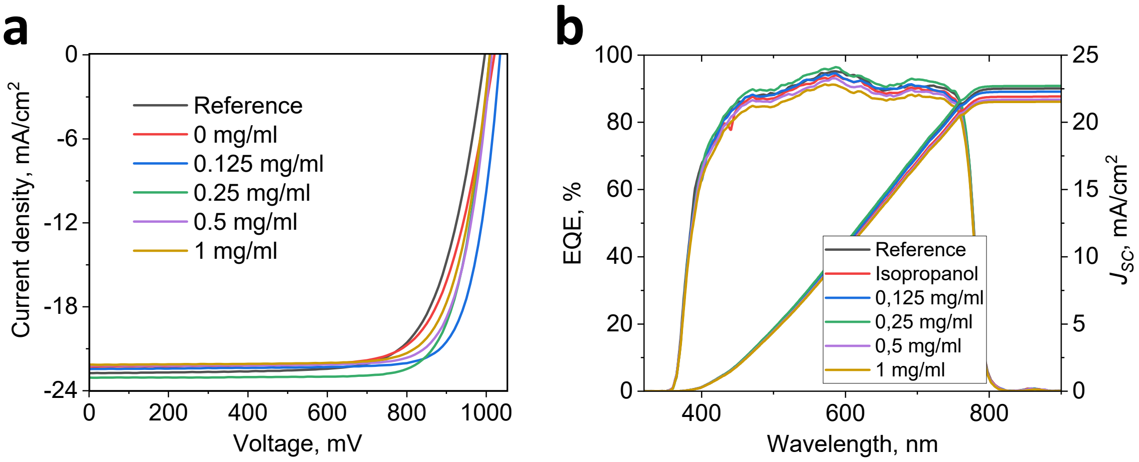

Figure S23. *J-V* curves (a) and EQE spectra (b) of perovskite solar cells with different concentrations of compound **1**

Table S6. Photovoltaic parameters of perovskite solar cells using compound **1** as interlayer*

Concentration of **1**, mg/ml	V_{OC}, mV	J_{SC}, mA/cm^2	FF, %	PCE, %
Reference	1003±18 (996)	22.3±0.5 (22.7)	72±3 (73)	15.5±1.0 (16.5)
0	999±21 (1020)	22.5±0.4 (22.1)	75±2 (74)	16.0±0.7 (16.7)
0.125	1019±28 (1035)	22.5±0.5 (22.5)	76±4 (80)	17.6±1.0 (18.6)
0.25	1002±19 (1009)	22.5±0.6 (23.0)	79±1 (79)	18.0±0.3 (18.3)
0.5	1014±11 (1014)	20.6±0.3 (22.2)	78±2 (79)	17.0±0.8 (17.8)
1	998±20 (1008)	21.3±0.8 (22.0)	76±3 (78)	17.0±0.3 (17.3)

* - Average parameters for a batch of 16 cells are given, while the champion cell characteristics are presented in brackets.

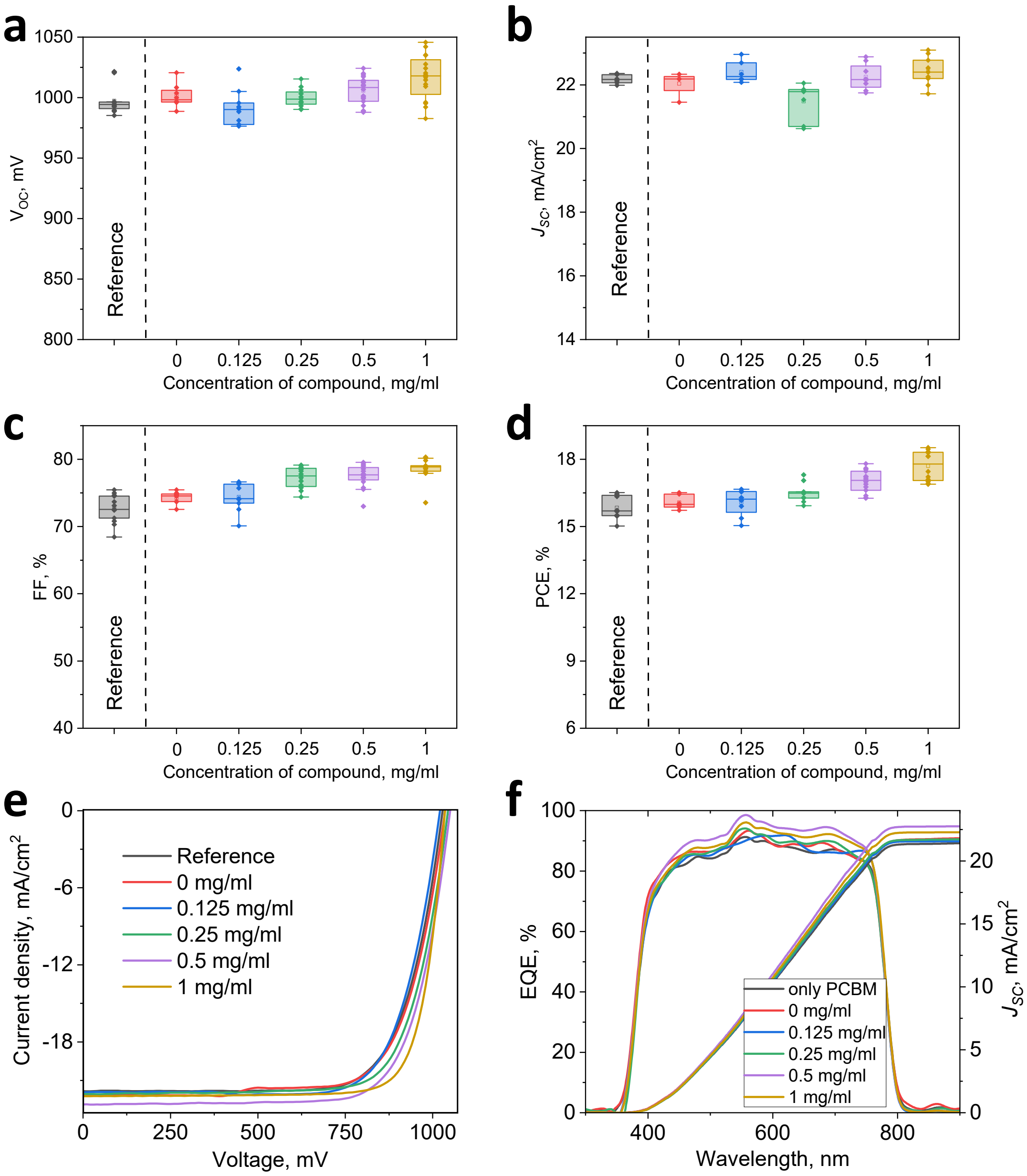

Figure S24. V_{OC} (a), J_{SC} (b), FF (c) and PCE (d) of PSCs as a function of concentration of **2**. *J*-V curves (e) and EQE (f) of the best devices

Table S7. Photovoltaic parameters of best solar cells with using of **2** as interlayer

Concentration of **2**, mg/ml	V_{OC}, mV	J_{SC}, mA/cm^2	FF, %	PCE, %
Reference	1002±29 (1027)	22.0±0.4 (21.7)	73±3 (74)	16.0±0.5 (16.5)
0	997±11 (1035)	21.3±1.0 (22.2)	73±1 (72)	16.3±0.3 (16.5)
0.125	985±10 (1021)	22.2±0.8 (22.0)	74±3 (74)	16.2±0.5 (16.6)
0.25	1007±9 (1044)	21.5±0.6 (22.1)	77±2 (75)	16.7±0.6 (17.3)
0.5	1037±17 (1050)	22.4±0.5 (22.9)	77±3 (74)	17.5±0.4 (17.8)
1	1033±12 (1040)	22.5±0.5 (22.2)	77±1 (80)	17.9±0.6 (18.5)

* - Average parameters for a batch of 16 cells are given, while the champion cell characteristics are presented in brackets.

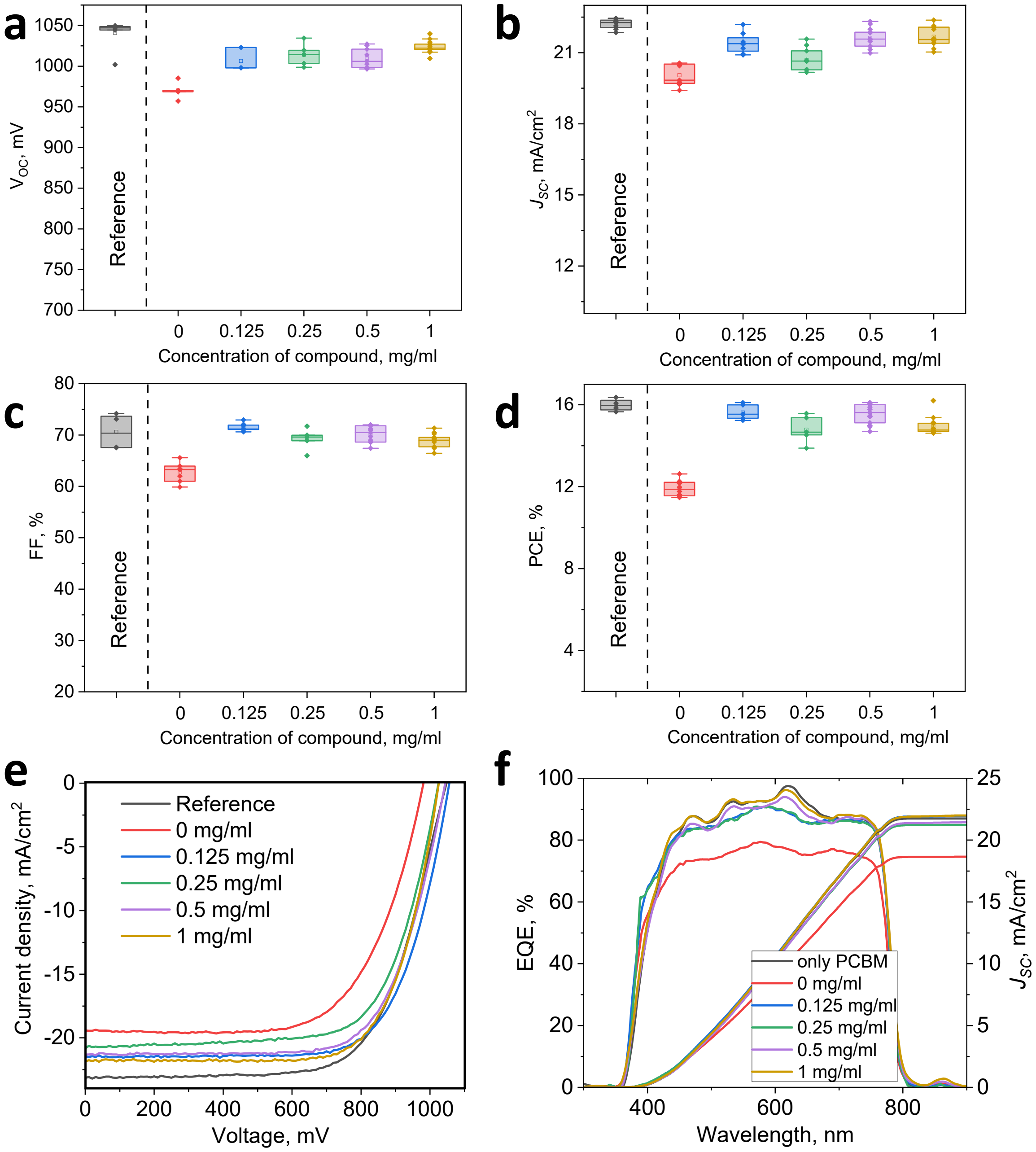

Figure S25. V_{OC} (a), J_{SC} (b), FF (c) and PCE (d) of PSCs as a function of concentration of **3**, *J*-V curves (e) and EQE (f) of the best devices

Table S8. Photovoltaic parameters of best solar cells with using of **3** as interlayer

Concentration of **3**, mg/ml	V_{OC}, mV	J_{SC}, mA/cm^2	FF, %	PCE, %
Reference	1034±17 (1045)	22.6±0.5 (23.1)	70±3 (68)	15.8±0.6 (16.4)
0	973±12 (981)	20.0±0.6 (19.4)	66±2 (66)	12.1±0.5 (12.6)
0.125	993±10 (1056)	21.9±0.3 (21.5)	71±2 (71)	15.5±0.6 (16.1)
0.25	1032±12 (1044)	21.0±0.6 (21.3)	69±3 (70)	15.0±0.6 (15.6)
0.5	1013±14 (1024)	22.0±0.3 (21.9)	69±3 (72)	15.8±0.3 (16.1)
1	1023±16 (1022)	22.0±0.3 (21.5)	72±2 (74)	15.8±0.5 (16.3)

* - Average parameters for a batch of 16 cells are given, while the champion cell characteristics are presented in brackets.

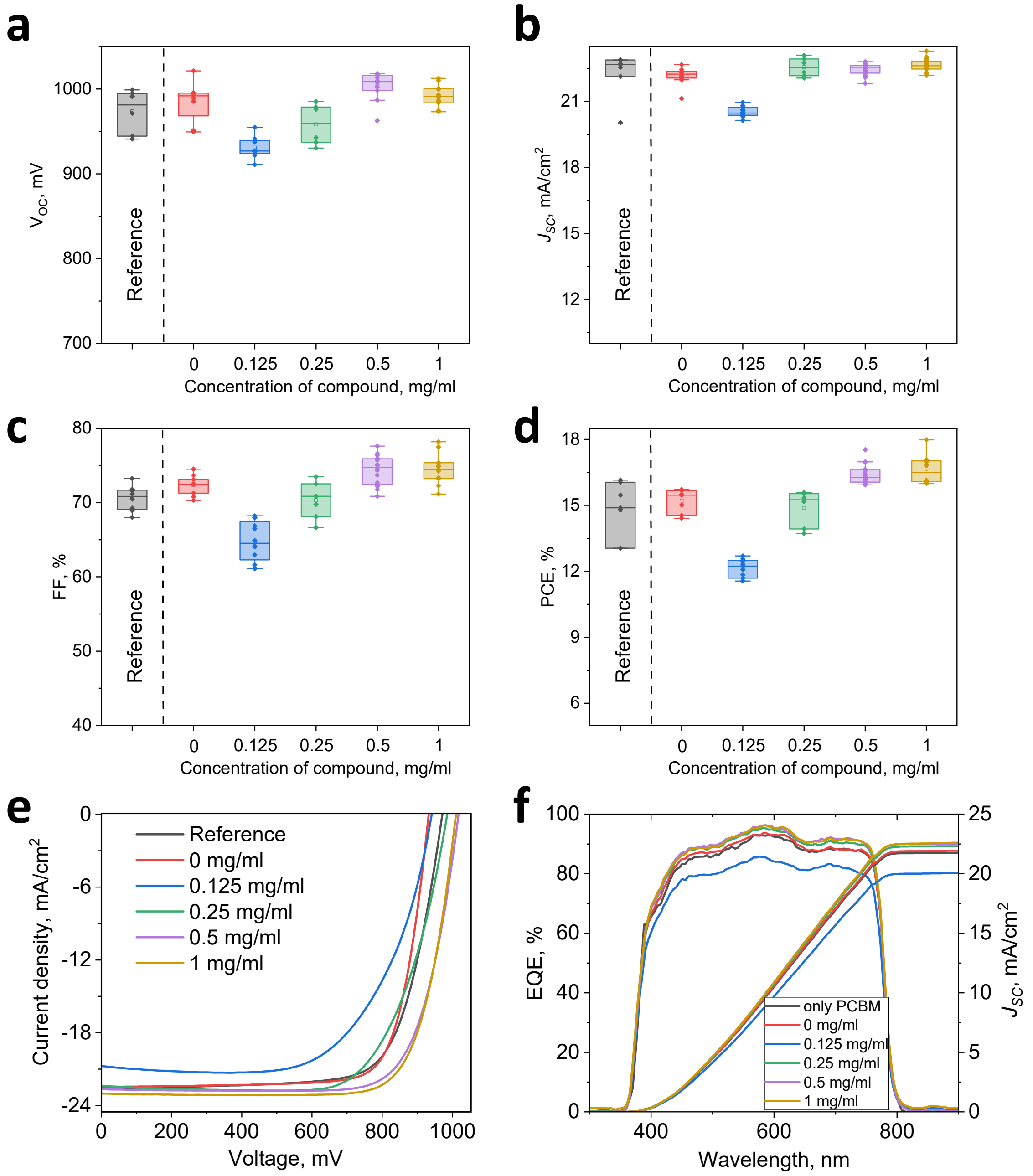

Figure S26. V_{OC} (a), J_{SC} (b), FF (c) and PCE (d) of PSCs as a function of concentration of **4**, *J*-V curves (e) and EQE (f) of the best devices

Table S9. Photovoltaic parameters of best solar cells with using of **4** as interlayer

Concentration of **4**, mg/ml	V_{OC}, mV	J_{SC}, mA/cm^2	FF, %	PCE, %
Reference	968±31 (972)	22.3±0.6 (22.7)	70±3 (73)	14.1±2.0 (16.1)
0	969±43 (930)	22.2±0.5 (22.3)	73±5 (78)	14.8±0.9 (16.2)
0.125	920±35 (941)	20.4±0.6 (20.8)	64±4 (65)	12.0±0.7 (12.7)
0.25	948±37 (985)	22.4±0.7 (22.3)	69±5 (71)	14.2±1.4 (15.6)
0.5	982±36 (1017)	22.5±0.3 (22.7)	74±4 (76)	16.1±1.5 (17.5)
1	999±23 (1015)	22.6±0.7 (23.0)	73±5 (77)	16.2±1.8 (18.0)

* - Average parameters for a batch of 16 cells are given, while the champion cell characteristics are presented in brackets.

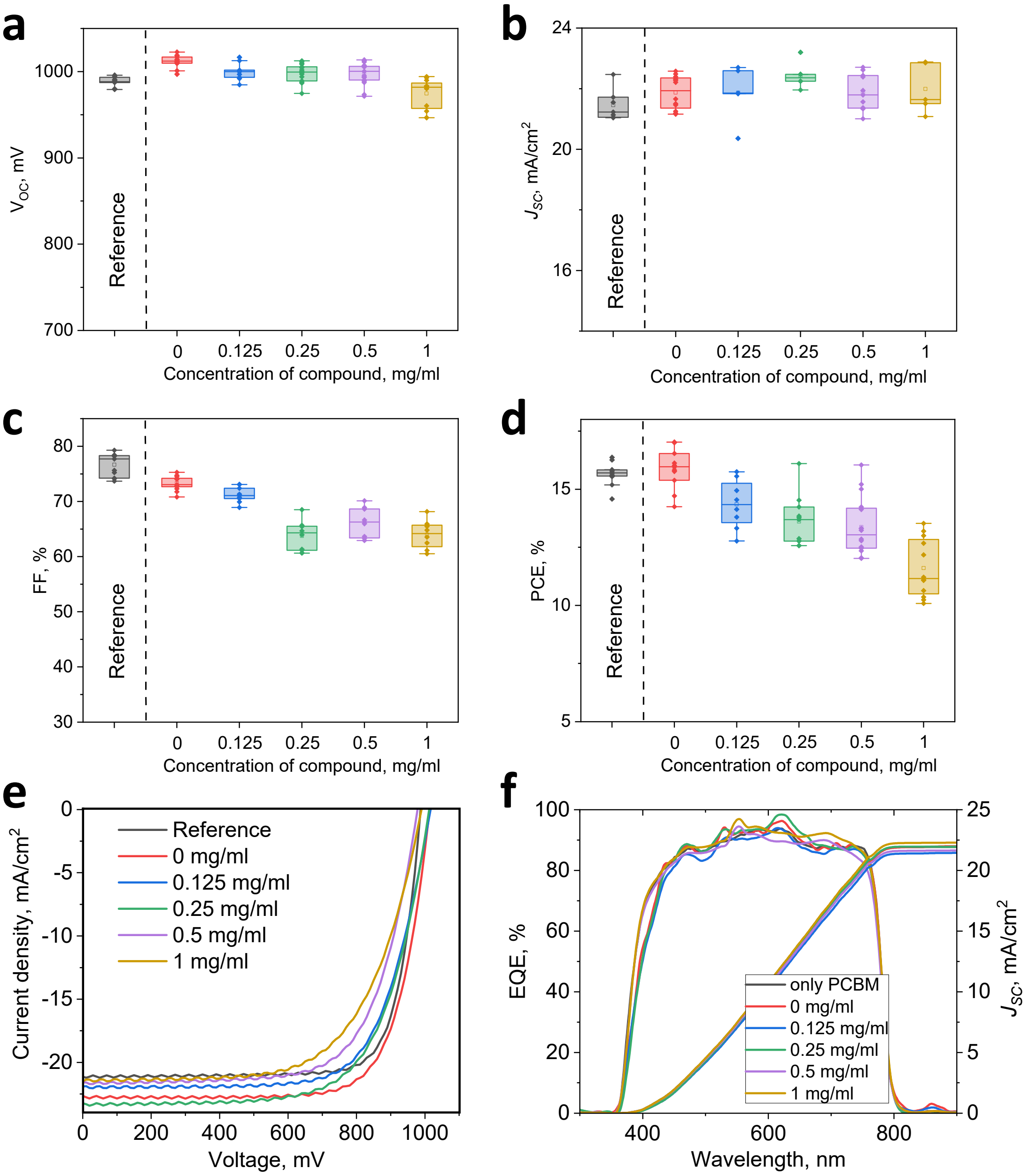

Figure S27. V_{OC} (a), J_{SC} (b), FF (c) and PCE (d) of PSCs as a function of concentration of **5**, *J*-V curves (e) and EQE (f) of the best devices

Table S10. Photovoltaic parameters of best solar cells with using of **5** as interlayer

Concentration of **5**, mg/ml	V_{OC}, mV	J_{SC}, mA/cm^2	FF, %	PCE, %
Reference	988±8 (989)	21.3±1.2 (21.2)	76±3 (78)	15.6±0.8 (16.4)
0	1012±11 (1014)	21.9±0.8 (22.7)	73±2 (75)	16.0±1.3 (17.3)
0.125	1004±13 (1016)	21.9±0.8 (21.8)	70±3 (71)	14.6±1.1 (15.7)
0.25	998±14 (1011)	21.9±1.3 (23.1)	64±5 (69)	14.6±1.5 (16.1)
0.5	1004±10 (978)	22.0±0.7 (21.6)	66±4 (69)	12.6±2.0 (14.6)
1	985±10 (989)	22.3±1.0 (21.4)	65±3 (64)	12.1±1.4 (13.5)

* - Average parameters for a batch of 16 cells are given, while the champion cell characteristics are presented in brackets.

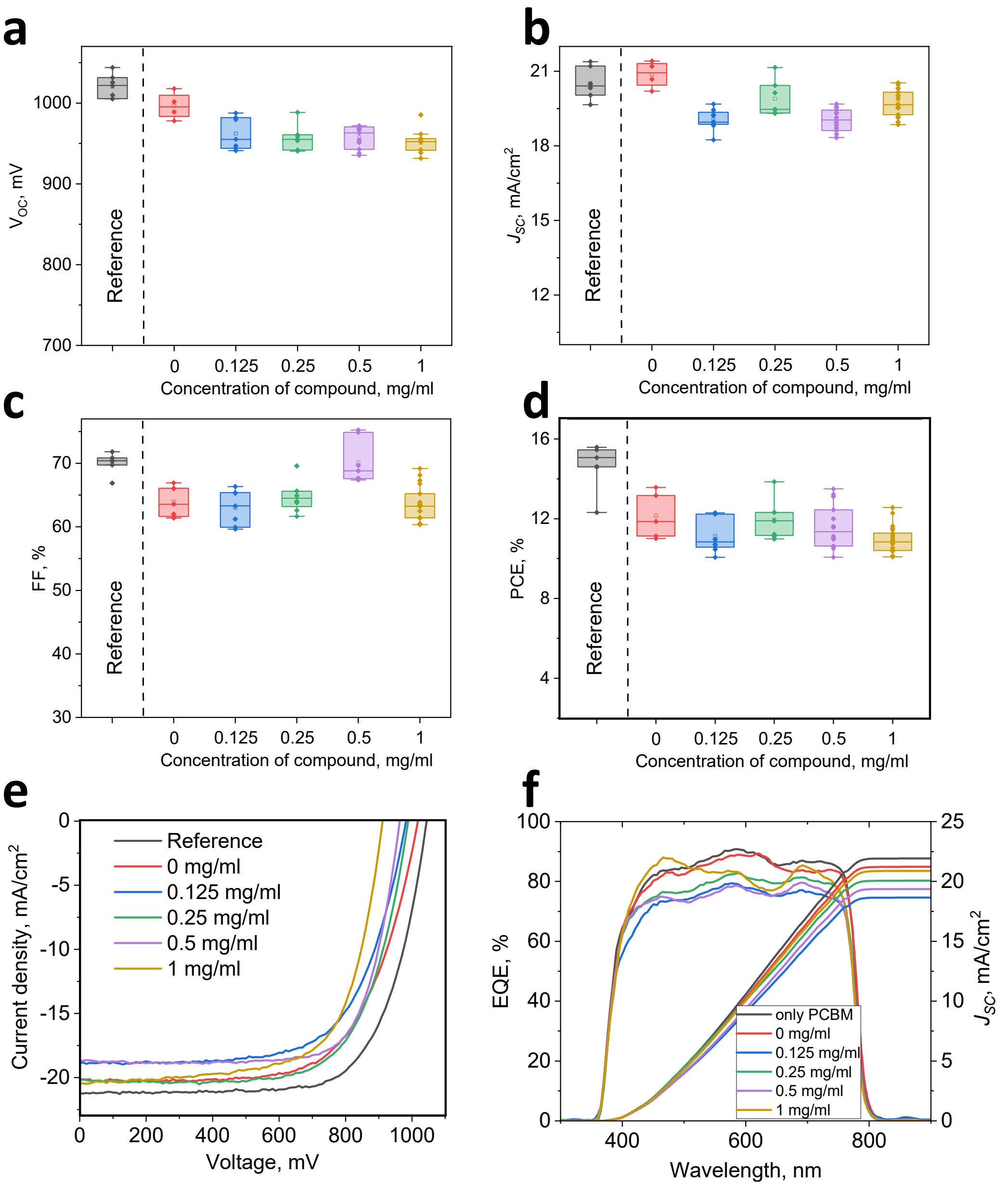

Figure S28. V_{OC} (a), J_{SC} (b), FF (c) and PCE (d) of PSCs as a function of concentration of **6**, *J*-V curves (e) and EQE (f) of the best devices

Table S11. Photovoltaic parameters of best solar cells with using of **6** as interlayer.

Concentration of **6**, mg/ml	V_{OC}, mV	J_{SC}, mA/cm^2	FF, %	PCE, %
Reference	1026±17 (1042)	20.4±1.0 (21.1)	69±3 (71)	13.6±2.0 (15.6)
0	1002±16 (1018)	19.7±1.7 (20.2)	64±3 (66)	12.2±1.4 (13.6)
0.125	970±18 (980)	19.0±0.7 (19.3)	61±4 (65)	10.7±1.6 (12.3)
0.25	972±16 (988)	20.3±0.9 (20.1)	66±4 (70)	12.1±1.7 (13.9)
0.5	959±14 (963)	18.7±1.0 (18.7)	72±4 (75)	11.8±1.7 (13.5)
1	966±20 (912)	20.1±1.0 (20.6)	65±4 (67)	11.2±1.5 (12.6)

* - Average parameters for a batch of 16 cells are given, while the champion cell characteristics are presented in brackets.

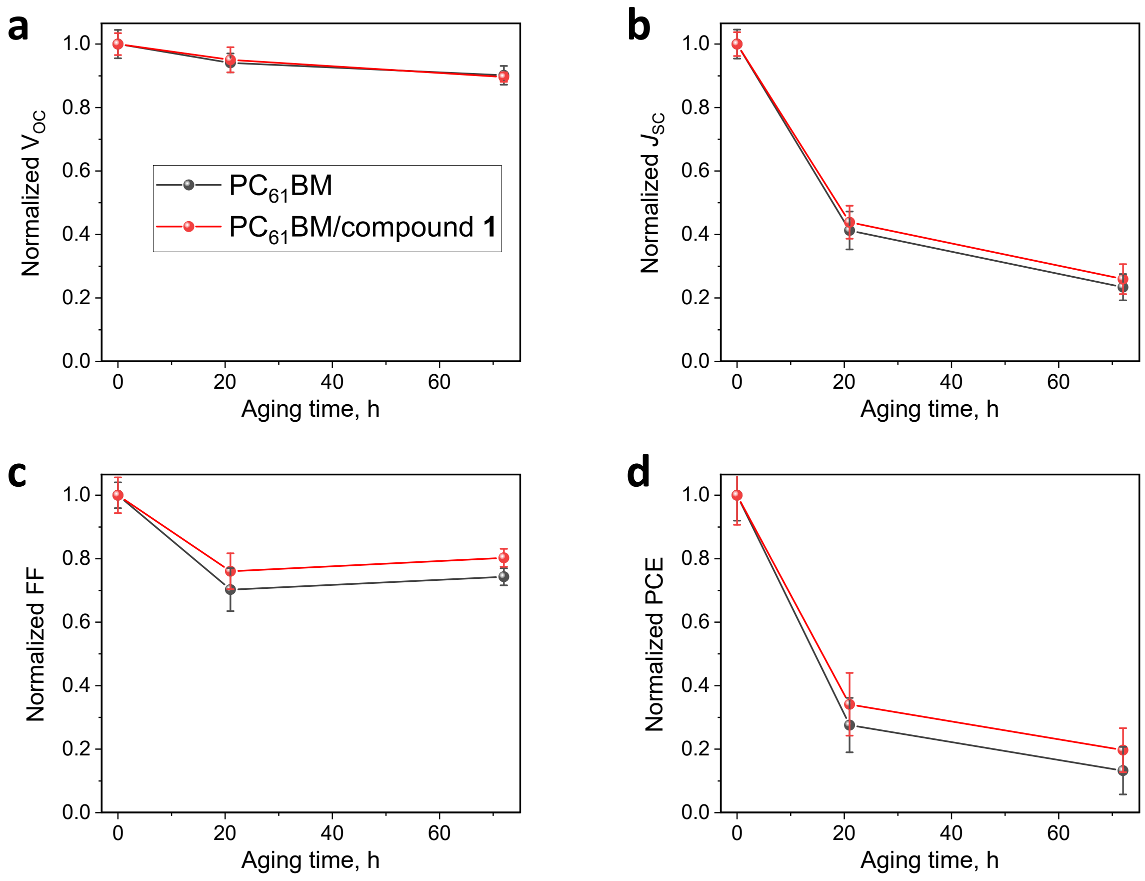

Figure S29. The evolution of the normalized open-circuit voltage (a), short-circuit current (b), fill factor (c) and power conversion efficiency (d) of perovskite solar cells using bare $PC_{61}BM$ and its combination with compound 1 as ETL materials.

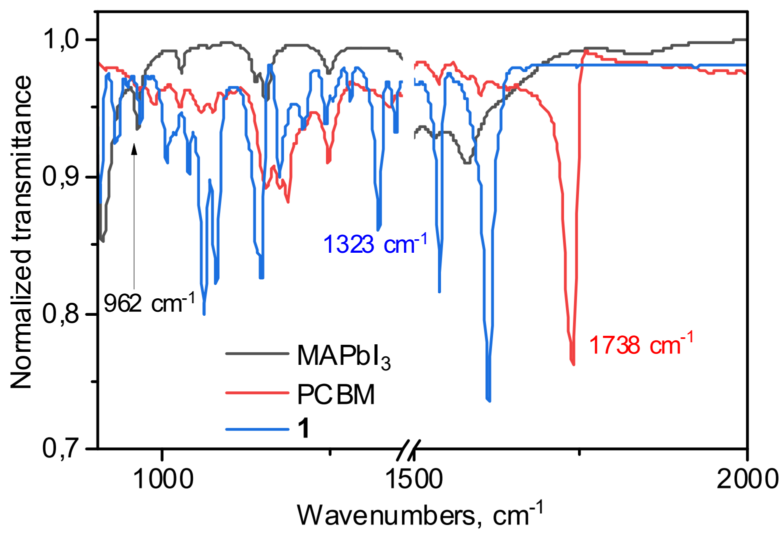

Figure S30. ATR FTIR spectra of $MAPbI_3$, $PC_{61}BM$, and **1**.

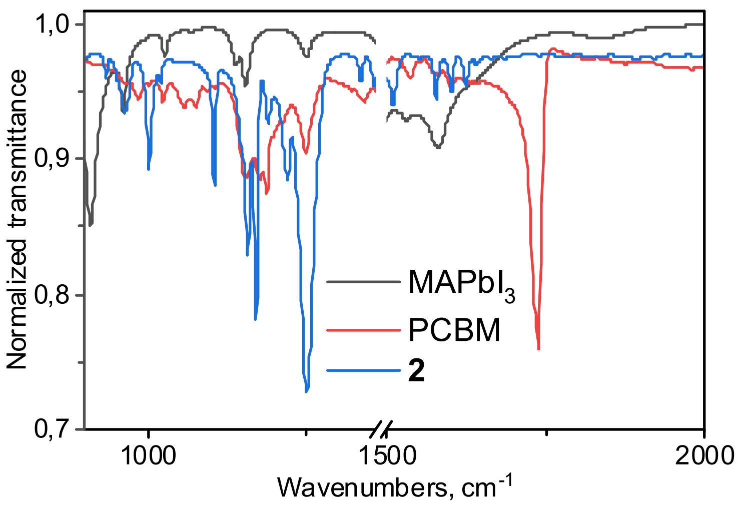

Figure S31. ATR FTIR spectra of $MAPbI_3$, $PC_{61}BM$, and **2**.

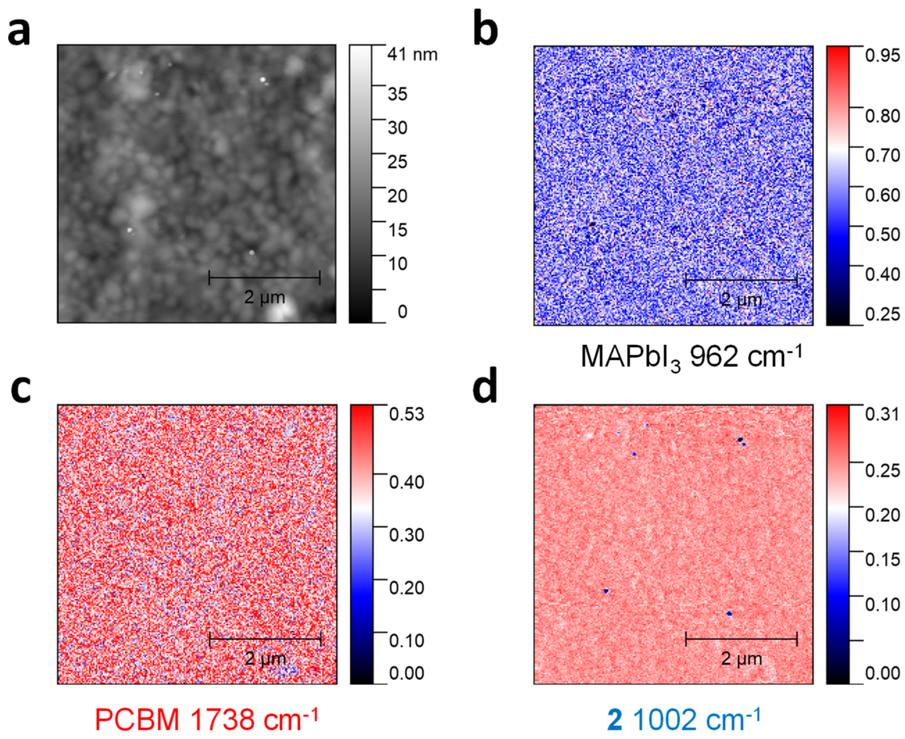

Figure S32. AFM topography of ITO/PTA/$MAPbI_3$/$PC_{61}BM$/**2** film (a); mappings of ITO/PTA/$MAPbI_3$/$PC_{61}BM$/**2** topography at frequencies of 962 cm^{-1} (b), 1738 cm^{-1} (c), and 1002 cm^{-1} (d), which are characteristic for $MAPbI_3$, $PC_{61}BM$, and **2**, respectively.

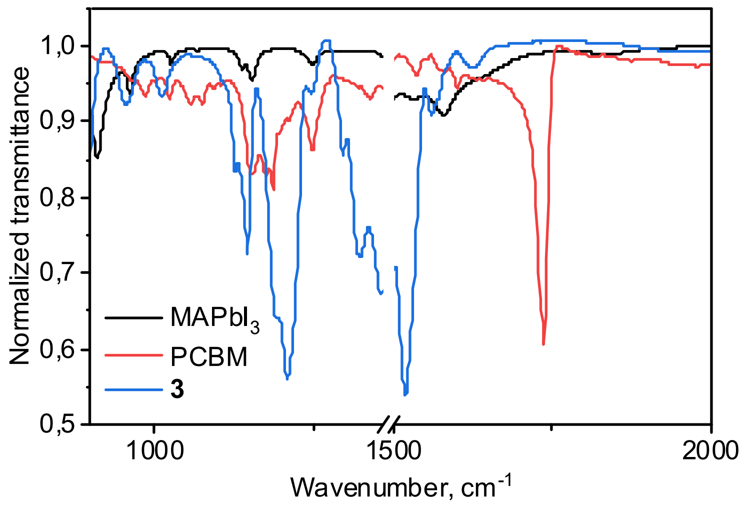

Figure S33. ATR FTIR spectra of $MAPbI_3$, $PC_{61}BM$, and **3**.

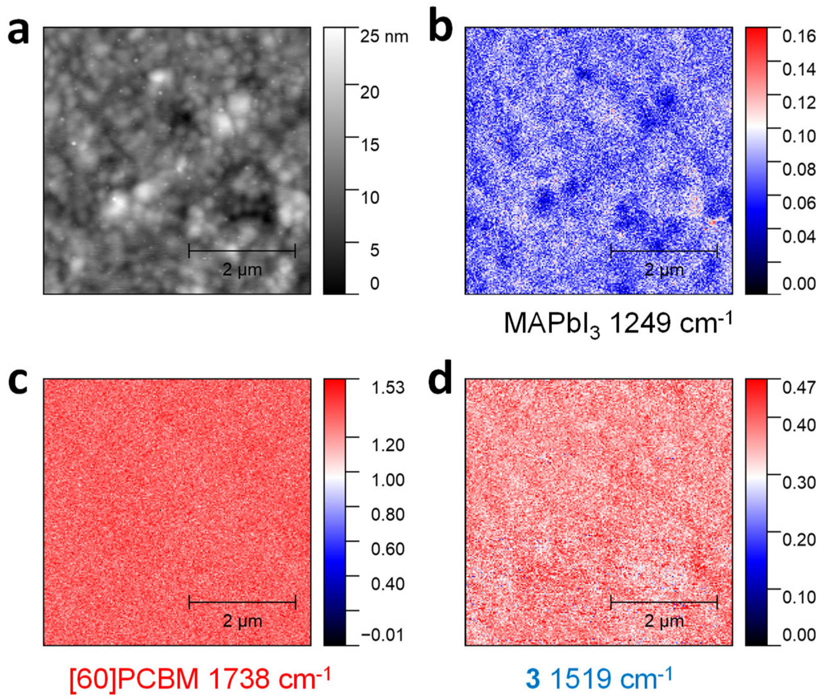

Figure S34. AFM topography of ITO/PTA/$MAPbI_3$/$PC_{61}BM$/**3** film (a); mappings of ITO/PTA/$MAPbI_3$/$PC_{61}BM$/**3** topography at frequencies of 1249 cm^{-1} (b), 1738 cm^{-1} (c), and 1519 cm^{-1} (d), which are characteristic for $MAPbI_3$, $PC_{61}BM$, and **3**, respectively.

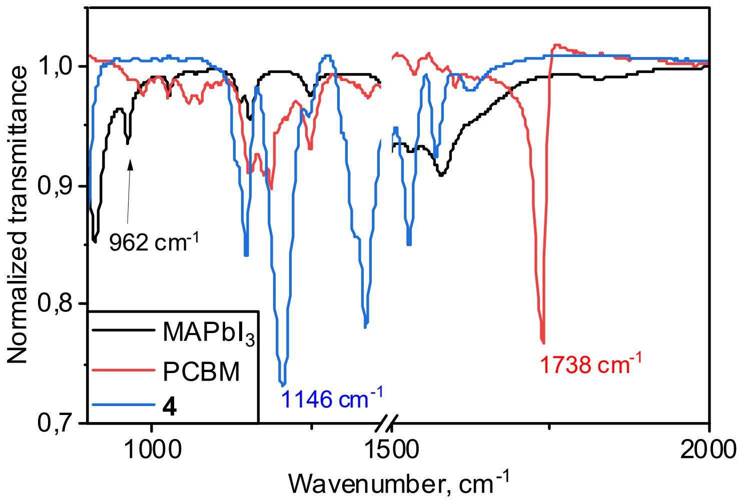

Figure S35. ATR FTIR spectra of $MAPbI_3$, $PC_{61}BM$, and **4**.

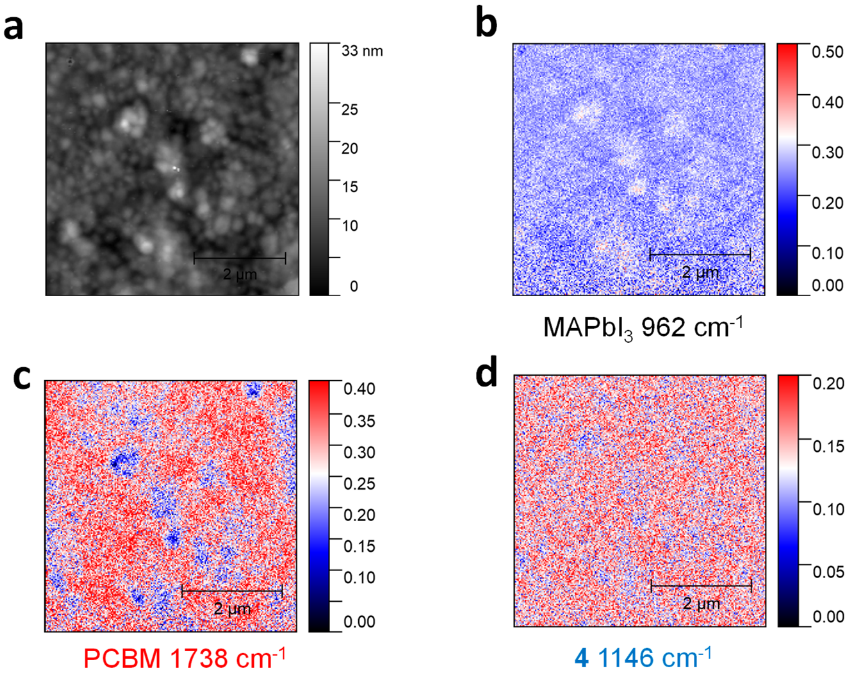

Figure S36. AFM topography of ITO/PTA/$MAPbI_3$/$PC_{61}BM$/**4** film (a); mappings of ITO/PTA/$MAPbI_3$/$PC_{61}BM$/**4** topography at frequencies of 962 cm^{-1} (b), 1738 cm^{-1} (c), and 1146 cm^{-1} (d), which are characteristic for **4**, $PC_{61}BM$, and $MAPbI_3$, respectively.

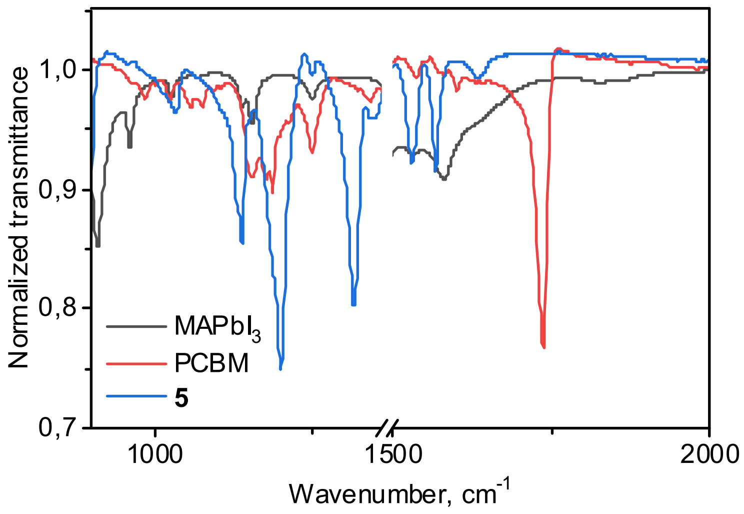

Figure S37. ATR FTIR spectra of $MAPbI_3$, $PC_{61}BM$, and **5**.

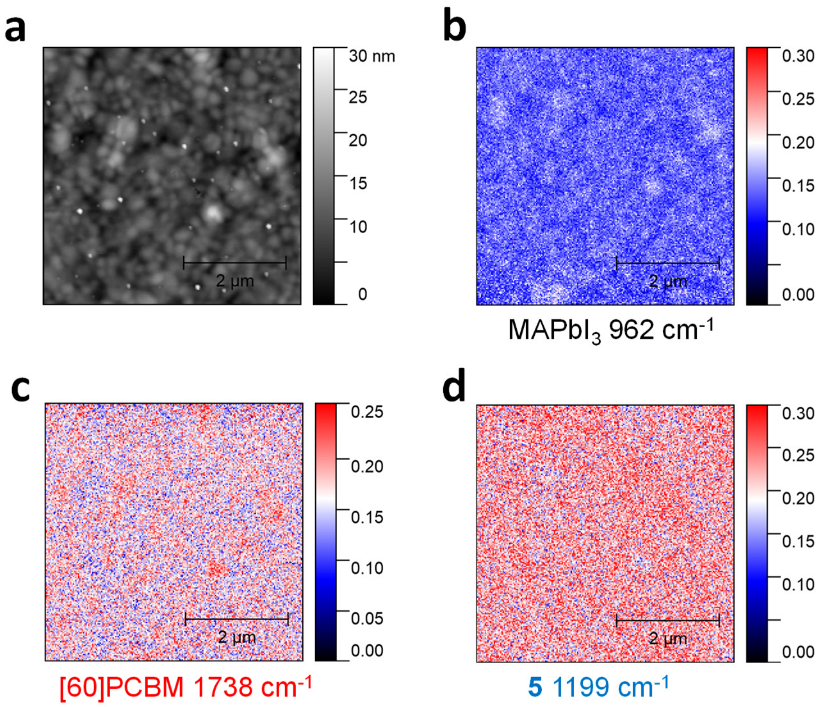

Figure S38. AFM topography of ITO/PTA/$MAPbI_3$/$PC_{61}BM$/**5** film (a); mappings of ITO/PTA/$MAPbI_3$/$PC_{61}BM$/**5** topography at frequencies of 962 cm^{-1} (b), 1738 cm^{-1} (c), and 1199 cm^{-1} (d), which are characteristic for **5**, $PC_{61}BM$, and $MAPbI_3$, respectively.

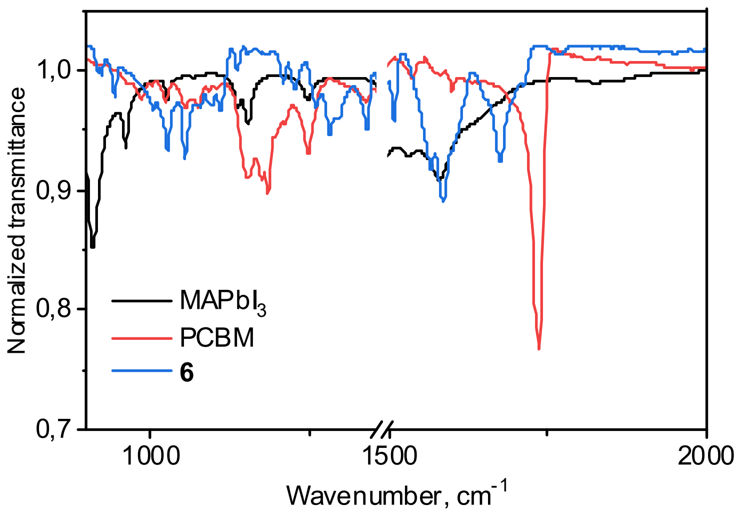

Figure S39. ATR FTIR spectra of $MAPbI_3$, $PC_{61}BM$, and **6**.

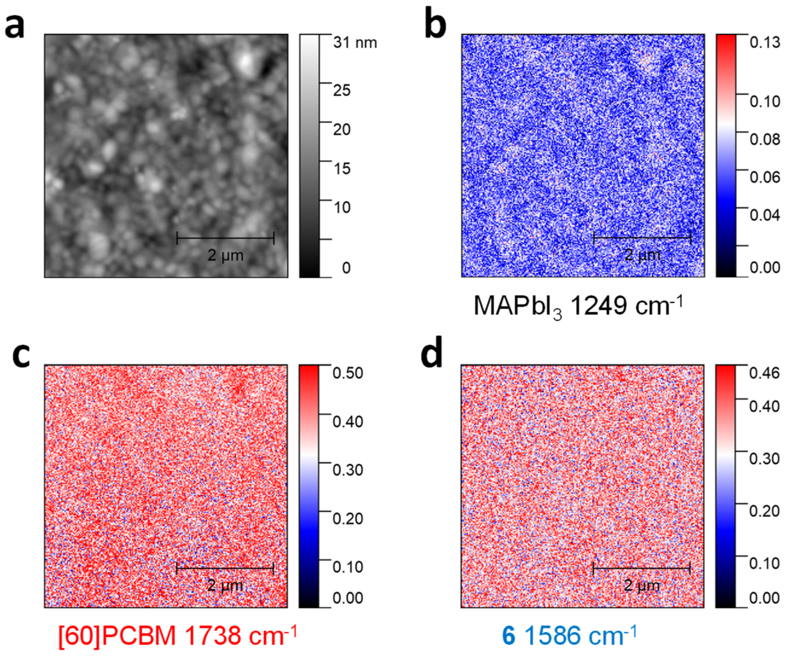

Figure S40. AFM topography of ITO/PTA/$MAPbI_3$/$PC_{61}BM$/**6** film (a); mappings of ITO/PTA/$MAPbI_3$/$PC_{61}BM$/**6** topography at frequencies of 962 cm^{-1} (b), 1738 cm^{-1} (c), and 1586 cm^{-1} (d), which are characteristic for **6**, $PC_{61}BM$, and $MAPbI_3$, respectively.
